# Clinical outcomes in a primary-level non-communicable disease programme for Syrian refugees and the host population in Jordan: A cohort analysis using routine data

**DOI:** 10.1371/journal.pmed.1003279

**Published:** 2021-01-11

**Authors:** Éimhín Ansbro, Tobias Homan, David Prieto Merino, Kiran Jobanputra, Jamil Qasem, Shoaib Muhammad, Taissir Fardous, Pablo Perel

**Affiliations:** 1 Centre for Global Chronic Conditions, London School of Hygiene & Tropical Medicine, London, United Kingdom; 2 Médecins Sans Frontières, London, United Kingdom; 3 Department of Non-communicable Disease Epidemiology, Faculty of Epidemiology and Population Health, London School of Hygiene &Tropical Medicine, London, United Kingdom; 4 Médecins Sans Frontières, Amman, Jordan; 5 Médecins Sans Frontières, Irbid, Jordan; 6 Health Economy Directorate, Ministry of Health of Jordan, Amman, Jordan; Columbia University Mailman School of Public Health, UNITED STATES

## Abstract

**Background:**

Little is known about the content or quality of non-communicable disease (NCD) care in humanitarian settings. Since 2014, Médecins Sans Frontières (MSF) has provided primary-level NCD services in Irbid, Jordan, targeting Syrian refugees and vulnerable Jordanians who struggle to access NCD care through the overburdened national health system. This retrospective cohort study explored programme and patient-level patterns in achievement of blood pressure and glycaemic control, patterns in treatment interruption, and the factors associated with these patterns.

**Methods and findings:**

The MSF multidisciplinary, primary-level NCD programme provided facility-based care for cardiovascular disease, diabetes, and chronic respiratory disease using context-adapted guidelines and generic medications. Generalist physicians managed patients with the support of family medicine specialists, nurses, health educators, pharmacists, and psychosocial and home care teams. Among the 5,045 patients enrolled between December 2014 and December 2017, 4,044 eligible adult patients were included in our analysis, of whom 72% (2,913) had hypertension and 63% (2,546) had type II diabetes. Using visits as the unit of analysis, we plotted the following on a monthly basis: mean blood pressure among hypertensive patients, mean fasting blood glucose and HbA1c among type II diabetic patients, the proportion of each group achieving control, mean days of delayed appointment attendance, and the proportion of patients experiencing a treatment interruption. Results are presented from programmatic and patient perspectives (using months since programme initiation and months since cohort entry/diagnosis, respectively). General linear mixed models explored factors associated with clinical control and with treatment interruption. Mean age was 58.5 years, and 60.1% (2,432) were women. Within the programme’s first 6 months, mean systolic blood pressure decreased by 12.4 mm Hg from 143.9 mm Hg (95% CI 140.9 to 146.9) to 131.5 mm Hg (95% CI 130.2 to 132.9) among hypertensive patients, while fasting glucose improved by 1.12 mmol/l, from 10.75 mmol/l (95% CI 10.04 to 11.47) to 9.63 mmol/l (95% CI 9.22 to 10.04), among type II diabetic patients. The probability of achieving treatment target in a visit was 63%–75% by end of 2017, improving with programme maturation but with notable seasonable variation. The probability of experiencing a treatment interruption declined as the programme matured and with patients’ length of time in the programme. Routine operational data proved useful in evaluating a humanitarian programme in a real-world setting, but were somewhat limited in terms of data quality and completeness. We used intermediate clinical outcomes proven to be strongly associated with hard clinical outcomes (such as death), since we had neither the data nor statistical power to measure hard outcomes.

**Conclusions:**

Good treatment outcomes and reasonable rates of treatment interruption were achieved in a multidisciplinary, primary-level NCD programme in Jordan. Our approach to using continuous programmatic data may be a feasible way for humanitarian organisations to account for the complex and dynamic nature of interventions in unstable humanitarian settings when undertaking routine monitoring and evaluation. We suggest that frequency of patient contact could be reduced without negatively impacting patient outcomes and that season should be taken into account in analysing programme performance.

## Abstract 2

Éimhín Ansbro and co-workers report on provision of care for non-communicable diseases in a humanitarian setting in Jordan.

## Author summary

**Why was this study done?:**

- Humanitarian actors have recently turned their attention to formally incorporating care for chronic non-communicable diseases (NCDs) into their programmes. NCDs include conditions such as hypertension (high blood pressure), heart disease, and diabetes.
- Little is known about the quality or effectiveness of current NCD programmes in humanitarian contexts, and more research is needed around how to deliver good-quality NCD care to people affected by humanitarian crises.

**What did the researchers do and find?:**

- Using routine clinic data, rather than research study data, we examined the levels of blood pressure and diabetes control as well as treatment interruption among a group of Syrian refugee and Jordanian patients attending a Médecins Sans Frontières (MSF) primary care clinic in north Jordan.
- Among 4,044 adult NCD patients eligible to be included in the study, 72% (2,913) had hypertension and 63% (2,546) had type II diabetes.
- Within both groups, disease control was achieved at about 63%–75% of monthly visits. Clinical control varied by season; better blood pressure control was achieved in the hot summer months. In addition, the chances of treatment interruption decreased both as the programme matured and as individual patients remained longer in the programme.

**What do these findings mean?:**

- Having taken account of multiple potential influencing factors in our analysis, we found that the MSF programme was achieving good clinical results compared to previous published literature.
- However, these good outcomes were achieved in the context of a complex and costly programme and using conservative clinical targets.
- We suggest that (1) similar results may be possible while reducing the frequency of patient visits, (2) our analysis approach may be useful for monitoring and evaluation of complex programmes in unstable environments, and (3) season should be taken into account when analysing NCD programme performance.

## Introduction

Now into its ninth year, the Syrian crisis continues to ravage the Syrian population. Since 2011, over 6.1 million people have been internally displaced, while over 6.6 million people have fled as refugees into the neighbouring countries Jordan, Lebanon, and Turkey [1]. Syria has passed through an epidemiological transition. Before the conflict, non-communicable diseases (NCDs) (mainly cardiovascular disease [CVD], cancer, chronic respiratory disease, and diabetes) caused more deaths than infectious disease and accounted for 77% of total mortality [2]. Host country and humanitarian health systems have had to adapt to new realities in responding to the Syrian crisis: tackling a high NCD burden and reaching a mainly urban-based (rather than camp-based) refugee population in the context of stressed local health systems [3–7].

Jordan hosts almost 670,000 United Nations High Commissioner for Refugees (UNHCR) registered Syrian refugees and, globally, ranks second only to Lebanon in number of refugees hosted relative to national population [1,8]. In addressing the rising NCD burden, Jordanian health policy has shifted focus from secondary- or tertiary-level NCD care to strengthening its primary NCD care delivery. Registered Syrian refugees are eligible to access Ministry of Health (MOH) primary care NCD services, but financial barriers, complex care pathways and referral systems, and limited health facility capacity have curtailed their access [7]. Jordan has enacted sequential humanitarian policy changes during the crisis, initially providing free, limited primary care to Syrian refugees, adding user fees in 2014, and then increasing user fees to the full ‘foreigner rate’ in early 2018 [9]. In response, Syrian refugees have shifted care seeking from the public sector towards non-governmental organisations (NGOs) and the private sector [10].

Since 2014, Médecins Sans Frontières (MSF), a humanitarian medical organisation, has provided primary-level NCD care for Syrian refugees and the host population at 2 clinics in Irbid, in north Jordan. Humanitarian actors’ programmatic experience in NCD care delivery has grown in the last decade due to the increasing global NCD burden, current conflicts in several middle-income countries of the Middle East, and the synergies between HIV, tuberculosis, and NCDs [11,12]. In response to the limited evidence, guidelines, and tools available to guide NCD interventions in low- and middle-income countries, especially for displaced, conflict-affected populations, humanitarian actors are developing NCD-specific clinical and operational guidance and monitoring and evaluation tools [13–16]. The recent discourse around quality of care and universal healthcare has underlined the importance of measuring quality and effectiveness as primary-level NCD care is scaled up in humanitarian settings and in low- and middle-income countries more broadly [17–20]. NCD programmes in humanitarian crises pose unique challenges for programme evaluation since they are often complex interventions that rapidly adapt to volatile contexts. Routine data may be limited and of poor quality, and accurate evaluation methods that involve minimal disruption to busy staff providing routine care are needed. Programmes with electronic health records, such as United Nations Relief and Works Agency for Palestinian Refugees (UNRWA) clinics and some MSF settings, have published descriptive cohort analyses of NCD cohorts involving quarterly or annual new registrations and outcomes reporting [21–23].

MSF undertook a mixed methods evaluation of the Irbid NCD programme using the RE-AIM implementation research framework [24]. In this paper, we explore the programme’s effectiveness, from both programmatic and patient perspectives. For this we modelled the per visit probability of achieving intermediate clinical targets, that is, blood pressure (BP) control in adults with hypertension (HTN) and glycaemic control in patients with diabetes mellitus type II (DM II). We also modelled the chances of treatment interruption. Finally, we explored which factors (including patient, programme, and contextual factors) may have affected the chances of a patient achieving clinical targets or of experiencing an interruption to their treatment.

Our specific objectives were to (1) describe patterns in the attainment of intermediate clinical targets (BP and glycaemic control) and treatment interruption, since the start of the programme; (2) describe patterns in the attainment of intermediate clinical targets (BP and glycaemic levels) and treatment interruption, from entry to the cohort (or from month of new diagnosis); and (3) explore factors associated with achievement of clinical outcomes and with treatment interruption.

## Methods

### Study design

This was a retrospective cohort study that included all adult patients (18+ years) with a diagnosis of HTN and/or DM II who entered the MSF NCD programme in Irbid, Jordan, between 14 December 2014 and 31 December 2017 (see S1 Protocol).

### Setting

The study was conducted in Irbid, the second largest city in Jordan, located 30 minutes south of the border with Syria. Irbid governorate hosts over 165,000 Syrian refugees, who are mostly urban-based and living amongst the host population [4]. MSF commenced an NCD programme within a MOH primary care facility in Irbid, in north Jordan, in December 2014. A second site in the city was opened within a local NGO clinic in April 2016. The MSF programme was vertical, operating in parallel to the pre-existing activities at each site rather than integrating with them. The cohort size was capped by MSF at approximately 4,000 for operational and cost reasons, and the service achieved coverage of about 23% of Syrian adult refugees with NCDs in Irbid governorate [9].

### Participants

To be enrolled in the programme a person had to have a medical indication and a vulnerability indication (Syrian refugee or vulnerable member of the Jordanian host population). Medical indications included a history of HTN, established CVD (angina, myocardial infarction, ischaemic stroke, transient ischaemic attack, peripheral vascular disease, congestive heart failure), diabetes mellitus type I or II, chronic obstructive pulmonary disease, asthma, or hypothyroidism. Vulnerability was defined as having refugee status, low income, and/or no Jordanian public health insurance (thus required to co-pay for MOH services). Both medical and vulnerability enrolment criteria changed over time, for example isolated hypothyroidism was removed as an enrolment criterion, and vulnerability criteria were adapted. There were no limitations to enrolment in terms of place of residence or age. For this paper, only data of patients with HTN and/or DM II, aged 18 years and older, were included in the analysis. Only patients with HTN—either with a previously known diagnosis at programme entry or newly diagnosed at enrolment or subsequent visits—contributed data to the BP control analyses. Similarly, only DM II patients contributed data to the glycaemic control analyses.

### Intervention

The MSF programme was a multidisciplinary, primary care model, which used context-adapted clinical guidelines, medications based on the MSF and World Health Organization (WHO) Essential Medicines List, and task sharing to nurses where appropriate. It served Syrian refugees and vulnerable members of the Jordanian host population. The programme evolved from initially providing medical consultation, health education, and behaviour change counselling to also including individual- and group-based mental health and psychosocial support (MHPSS), a home visit service for house-bound patients, a humanitarian support worker providing social work services, and physiotherapy services. Care was provided by non-specialist doctors, supported by a family medicine specialist at each clinic, along with a team of nurses, trained health educators, psychosocial counsellors, pharmacists, physiotherapists, a social worker, and a home care team. Programmatic changes introduced during the study period included the initiation of task sharing to nurses of follow-up consultations for stable patients and the introduction of formal MSF NCD guidelines (S2 Fig).

Most patients were enrolled during the first 6 months of the programme (S2 Fig). The majority (over 90%) presented with established, self-reported diagnoses and were already on treatment at enrolment; measurements and new diagnoses were made based on the MSF NCD guidelines [16]. For hypertensive patients, BP was measured at each visit, and capillary fasting blood glucose (FBG) was checked annually. For diabetic patients, BP and FBG were measured at each visit, while glycosylated haemoglobin (HbA1c) was measured by an external laboratory every 3 months. At the first visit, doctors recorded a complete past medical, medication, and family medical history and performed a clinical examination. In addition, lifestyle CVD risk factors (smoking status, alcohol intake, exercise levels) were recorded, the global cardiovascular risk score was calculated using WHO CVD risk prediction charts, acute complications were identified and treated, long-term medications were prescribed for symptom management and secondary prevention of complications, patients were referred for laboratory testing as appropriate, and a follow-up interval was determined [25].

Follow-up visits involved reviewing patients’ symptoms and disease control, vital signs, and laboratory results; determining and recording new diagnoses; adjusting and/or initiating medications; and referring for further laboratory tests or to other health providers, as required. Health promoters provided individually tailored health education at each clinical contact. At enrolment, the doctor and health educator saw patients for 30 minutes each; patients on return visits spent approximately 15 minutes each with a clinician (either a doctor or nurse) and a health educator. By the end of 2017, nurses were performing 6% of follow-up consultations for stable patients, with doctors continuing to initiate and adjust all patients’ medications.

### Variables

The main outcomes of interest were HTN control (<140/90 mm Hg) and glycaemic control (capillary FBG ≤ 180 mg/dl [10.1 mmol/l] or HbA1c < 8% [46 mmol/mol]). MSF and other humanitarian actors have taken a consensus-based approach to developing guidelines, based on international best practice. They adopted a more conservative HbA1c target than the international norm of 7% (53 mmol/mol) to avoid hypoglycaemia in insulin-treated patients (who usually have no means to self-monitor in crisis settings) and used the same targets for all patients, irrespective of age or comorbidity, to simplify clinical guidelines and delivery [21,23,26]. Both FBG and HbA1c were used in this analysis since the FBG data were more complete and the sensitivity of HbA1c appears to be reduced in Arab populations [27].

Treatment delay was defined as the difference in days between the planned next appointment date and the actual next visit date. Treatment interruption was defined as a treatment delay greater than 31 days. If the planned appointment date was not registered, we assumed this was 1 month from the previous visit if the patient was not achieving clinical targets at that visit, and 3 months from the previous visit if the patient was achieving targets. We used the variables ‘treatment delay’ and ‘treatment interruption’, rather than the operational definition of ‘treatment defaulter’ included in our protocol, in order to capture periods of treatment interruption followed by a return to care (S1 Protocol).

### Data collection and management

Routine clinical data were maintained in paper-based, purpose-designed chronic care files, stored securely at each clinic. On a weekly basis, MSF data clerks entered data into a password-protected macro-based Excel software database specifically developed for the NCD programme. Data from all patients aged 18 years and older with a new or established diagnosis of HTN or DM II enrolled from December 2014 through to December 2017 in either of MSF’s NCD clinics in Irbid were included. Data from both clinics were aggregated and analysed using the statistical software R version 3.6.1 (2019-07-05).

### Data analysis and reporting

We used visits as the unit of analysis when analysing the main study outcomes. This allowed us to include all data from all patients, irrespective of their frequency or duration of attendance or whether they had missed appointments. It also accounted for the fact that BP and glycaemic control may vary from appointment to appointment.

Descriptive statistics were used to explore patient demographics at baseline and among those who remained in care at 6 and/or 12 months post-enrolment.

To examine control from the programmatic perspective, we plotted the monthly means across visits of systolic BP (SBP), the glycaemic variables (FBG and HbA1c), and the days of treatment delay as defined above, for each month from December 2014 to December 2017. We also plotted, for each month, the proportion of visits achieving BP and glycaemic control and the proportion of visits after which there was an interruption of treatment (i.e., delay to next visit of >31 days after the planned appointment).

To examine control from the patient perspective, we calculated the same means and proportions in periods of 30 days from disease identification (enrolment visit for those with pre-existing diagnoses, or the visit when a new diagnosis was made).

To explore factors associated with the mean levels of SBP, FBG, and HbA1c; control of BP, FBG, and HbA1c; and treatment delay/interruption at a given visit, we used 8 generalised linear mixed-effects models (GLMMs). For each of these outcomes, 2 models were adjusted: (1) a linear model for the continuous level of the outcome and (2) a logistic model to estimate the probability of the clinical target being reached or a treatment interruption occurring, as defined above. A random effect coefficient was included to adjust for repeated patient visits. The variables included in the models were time since patient’s diagnosis, time since beginning of the programme, sex, age, Syrian versus other nationality, diabetes status (for models exploring HTN control), HTN status (for models exploring glycaemic control), number of relevant NCD conditions, number of MSF-prescribed NCD medications, history of previous treatment interruption, and month of the year as a categorical variable (to account for seasonality). The specific statistical models were not pre-specified. We have reported our results in accordance with the STROBE checklist (S1 STROBE Checklist).

The London School of Hygiene & Tropical Medicine Ethics Review Committee (reference: 12239), the Médecins Sans Frontières Ethics Review Board, and the Jordanian MOH approved this study. Consent was not obtained from the study participants as these routinely collected programmatic data were analysed anonymously.

## Results

From December 2014 to December 2017, 5,045 patients attended an enrolment visit at the MSF NCD programme in Irbid, of whom 4,729 adult patients had an NCD targeted by the programme and were actually enrolled. Among the 4,044 patients with HTN and/or DM II (therefore, eligible for inclusion in the study), 2,913 (72%) had HTN and 2,546 (63%) had DM II at baseline, while 1,530 (32.4%) had both diagnoses. Among those with HTN, 92.7% were hypertensive at enrolment, and 7.3% were newly diagnosed at their first or subsequent visits; the proportions were similar for DM II (90.8% at first visit and 9.2% at subsequent visits).

Table 1 presents HTN/DM II patients’ socio-demographics, cardiovascular risk factors, and targeted NCD diagnoses (1) at enrolment, (2) among those who continued in care after 6 months, and (3) among those who continued in care after 12 months. Most were female (*n =* 2,432, 60.1%) and aged 41 years or older (mean = 58.5 years, SD = 11.6). Syrians made up 71.2% of included patients, while the remainder were mostly Jordanian. Of those with available data, most had either primary-level or no formal education (*n =* 1,220, 30.3%, and *n =* 672, 16.6%, respectively), and many lived in households of 7 or more people (*n =* 1,590, 39.1%). Over one-tenth (*n =* 446, 11.0%) defined themselves as mobility impaired, almost a quarter as active smokers (*n =* 946, 23.4%), and only around a third (*n =* 1,493; 36.9%) as physically active at enrolment. Over 80% (*n = *3,341, 82.6%) of included patients attended a 6-month follow-up appointment, while 76.9% (*n =* 3,109) attended a 12-month appointment. The demographic profile of those attending was similar at baseline, 6 months, and 12 months.

**Table 1:** Demographics, CVD risk factors, and NCD diagnoses among adult patients with hypertension and/or diabetes type II at enrolment and among those with visits at 6 and 12 months.

Variable	Baseline* N = 4,044		Patients returning at 6 months^ *N =* 3,341		Patients returning at 12 months^#^ *N =* 3,109	
	*n*	Percent	*n*	Percent or *p*-value	*n*	Percent or *p*-value
**Age group (years)**				*p =* 0.665		*p =* 0.688
18–40	243	6.0	174	5.2	164	5.3
41–65	2,690	66.5	2,244	67.2	2,099	67.5
66–80	987	24.4	824	24.7	758	24.4
>80	121	3.0	96	2.9	86	2.8
No data	3	0.1	3	0.1	2	0.1
**Sex**				*p =* 0.651		*p =* 0.947
Female	2,432	60.1	1,991	59.6	1,873	60.2
Male	1,612	39.9	1,350	40.4	1,236	39.8
**Syrian**				*p =* 0.342		*p =* 0.064
No	1,166	28.8	998	29.9	960	30.9
Yes	2,878	71.2	2,343	70.1	2,149	69.1
**Education**				*p =* 0.018		*p* < 0.001
No data	1,265	31.3	932	27.9	768	24.7
None	672	16.6	580	17.4	553	17.8
Primary	1,220	30.2	1,052	31.5	1,032	33.2
Secondary/higher	887	21.9	777	23.3	756	24.3
**Household size**				*p =* 0.721		*p =* 0.185
No data	281	6.9	217	6.5	179	5.8
1–3	792	19.6	672	20.1	629	20.2
4–7	1,391	34.4	1,172	35.1	1,100	35.4
>7	1,580	39.1	1,280	38.3	1,201	38.6
**Impaired mobility**				*p =* 0.508		*p =* 0.408
No data	123	3.0	87	2.6	80	2.6
No	3,475	85.9	2,891	86.5	2,699	86.8
Yes	446	11.0	363	10.9	330	10.6
**Smoker**				*p =* 0.494		*p =* 0.517
No data	15	0.4	6	0.2	6	0.2
Never smoked	2,511	62.1	2,078	62.2	1,953	62.8
Ex-smoker	572	14.1	478	14.3	440	14.2
Current smoker	946	23.4	779	23.3	710	22.8
**Exercise**				*p =* 0.997		*p =* 0.988
No data	228	5.6	190	5.7	177	5.7
Inactive	750	18.5	614	18.4	571	18.4
Moderate	1,573	38.9	1,306	39.1	1,221	39.3
Active	1,493	36.9	1,231	36.8	1,140	36.7
**Hypertension**				*p =* 0.984		*p =* 0.961
No	1,131	28.0	936	28.0	872	28.0
Yes	2,913	72.0	2,405	72.0	2,237	72.0
**Diabetes type II**				*p =* 0.839		*p =* 0.850
No	1,498	37.0	1,229	36.8	1,144	36.8
Yes	2,546	63.0	2,112	63.2	1,965	63.2
**Diabetes type I**				*p =* 0.898		*p =* 0.722
No	4,023	99.5	3,322	99.4	3,090	99.4
Yes	21	0.5	19	0.6	19	0.6
**CVD**				*p =* 0.483		*p =* 0.241
No	3,345	82.7	2,785	83.4	2,605	83.8
Yes	699	17.3	556	16.6	504	16.2
**Asthma**				*p =* 0.491		*p =* 0.540
No	3,914	96.8	3,223	96.5	3,000	96.5
Yes	130	3.2	118	3.5	109	3.5
**COPD**				*p =* 0.491		*p =* 0.680
No	4,008	99.1	3,317	99.3	3,085	99.2
Yes	36	0.9	24	0.7	24	0.8

*p*-Values compare distributions at 6 and 12 months with baseline distribution for each factor. Patients may have experienced treatment interruptions before returning at either 6 months or 12 months, and the group returning at 12 months may contain patients who did not attend at 6 months.

*Refers to proportion of total eligible adult patients with hypertension/type II diabetes.

^Proportion of enrolled patients in each category returning at 6 months (±30 days).

^#^Proportion of enrolled patients in each category returning at 12 months (±30 days).

COPD, chronic obstructive pulmonary disease; CVD, cardiovascular disease; NCD, non-communicable disease.

### Programmatic performance

**Fig 1:**
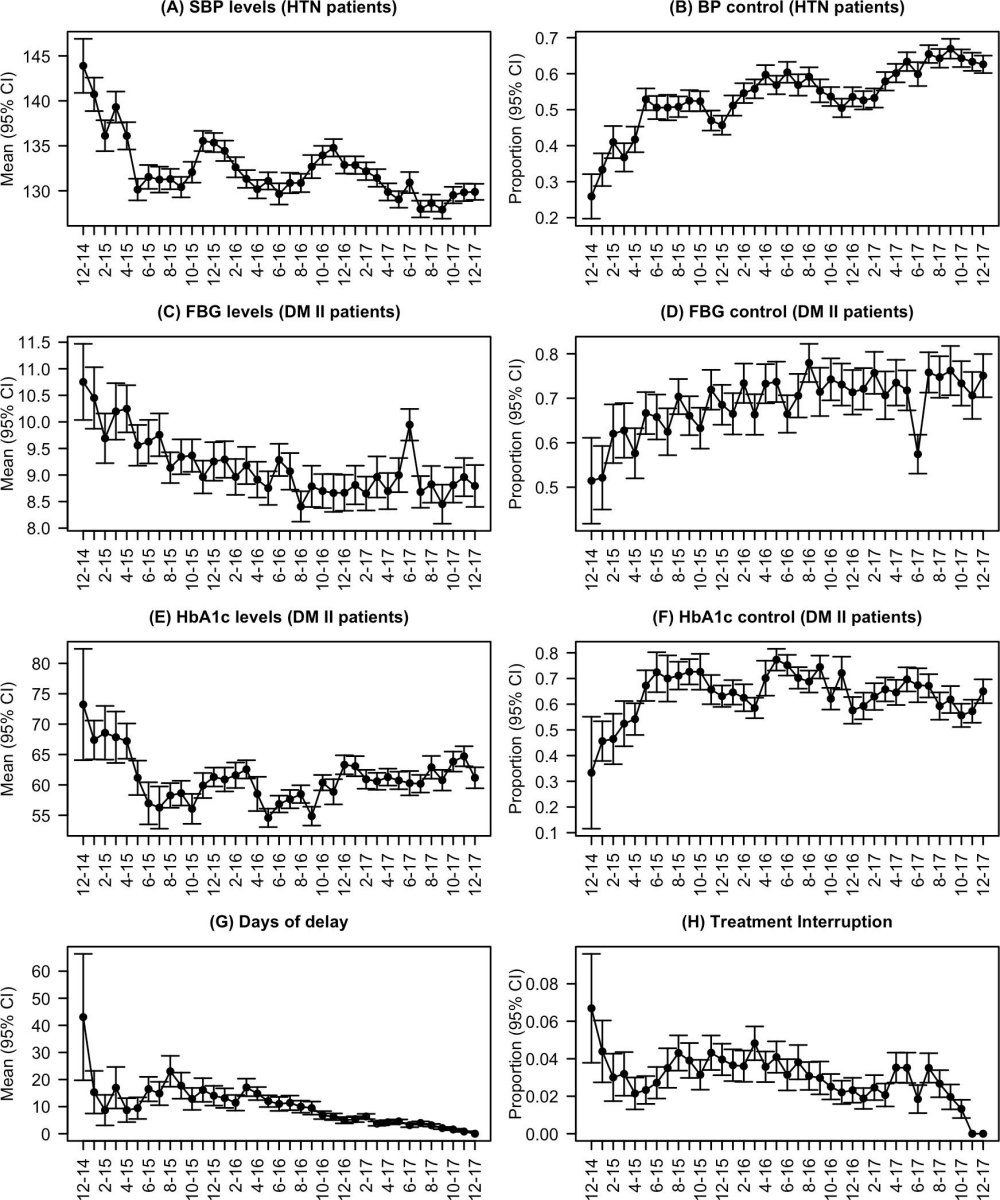
Programmatic patterns in achievement of clinical outcomes and in treatment interruption. (A) Mean monthly systolic blood pressure (SBP; mm Hg) for all visits of patients with hypertension (HTN). (B) Proportion of visits per month of patients with hypertension at which BP control was achieved. (C) Mean monthly fasting blood glucose (FBG) value (mmol/l) for all visits of patients with diabetes mellitus type II (DM II). (D) Proportion of visits per month of patients with diabetes type II at which FBG target was achieved. (E) Mean monthly glycosylated haemoglobin (HbA1c) value (mmol/mol) for all visits of patients with diabetes type II. (F) Proportion of visits per month of patients with diabetes type II at which HbA1c target was achieved. (G) Mean monthly days of delay (number of days after planned next visit that next visit actually occurred) for all visits of patients with hypertension and/or diabetes type II. (H) Proportion of visits per month of patients with hypertension and/or diabetes type II at which a treatment interruption occurred (next visit was >31 days after next planned visit date). Each panel reports the average per visit value, using visits as the unit of analysis, for each month since the start of the programme (December 2014 to December 2017 inclusive). BP control was defined as BP < 140/90 mm Hg; control targets in patients with type II diabetes were capillary FBG ≤ 180 mg/dl (10.1 mmol/l) or HbA1c < 8% (46 mmol/mol).

Fig 1 shows patterns in the achievement of treatment targets and in the occurrence of treatment interruption since the programme began in December 2014.

#### Hypertension

Among patients with HTN, mean per visit SBP decreased by 12.4 mm Hg in the programme’s first 6 months, from a mean of 143.9 mm Hg (95% CI 140.9 to 146.9) in December 2014 to 131.5 (95% CI 130.2 to 132.9) at 6 months, and by a further 1.6 mm Hg by the end of the study period, with a mean of 129.9 mm Hg (95% CI 128.9 to 130.8) in December 2017 (Fig 1A). There appeared to be seasonal variation as SBP increased annually by 4 to 5 mm Hg during the winter months (October to December).

Rates of control similarly improved from only 26% (95% CI 20% to 32%) in the first month, with a seasonal pattern. About 51%–60% of visits achieved BP targets in summer, decreasing by about 6% to 46%–54% in winter (Fig 1B).

#### Diabetes

Among patients with DM II, mean per visit FBG similarly decreased from the programmatic perspective, by 1.12 mmol/l, from 10.75 mmol/l (95% CI 10.04 to 11.47) in December 2014 to 9.63 mmol/l (95% CI 9.22 to 10.04) at 6 months, and by a further 0.37 mmol/l to 9.26 mmol/l (95% CI 8.89 to 9.62) at 12 months (Fig 1C), which mirrors the increasing proportion of visits achieving control over time since the programme began (Fig 1D). HbA1c control improved markedly during the programme course, from a mean of 73.23 mmol/mol (95% CI 64.07 to 82.39) in month 1, with approximately 33% (95% CI 12% to 55%) of DM II patients achieving target, to a mean of 56.97 mmol/mol (95% CI 53.50 to 60.43), with 72% (95% CI 65% to 80%) achieving control after the first 6 months of operation (Fig 1E and 1F). FBG control seemed to worsen during the month of June, especially in 2017. HbA1c control appeared to show a similar pattern of seasonality, with control deteriorating in the winter and improving in the summer.

#### Treatment interruption

The mean monthly days of delay following the next planned appointment fell from 43 days (95% CI 20 to 66) in December 2014 to 14 days (95% CI 10 to 18) 12 months later and to only 3 days in December 2017. The probability of treatment interruption also showed a downward pattern, dropping from 0.07 (95% CI 0.04 to 0.10) in the first month to 0.04 (95% CI 0.03 to 0.05) 12 months later.

### Individual (closed) cohort analysis

**Fig 2:**
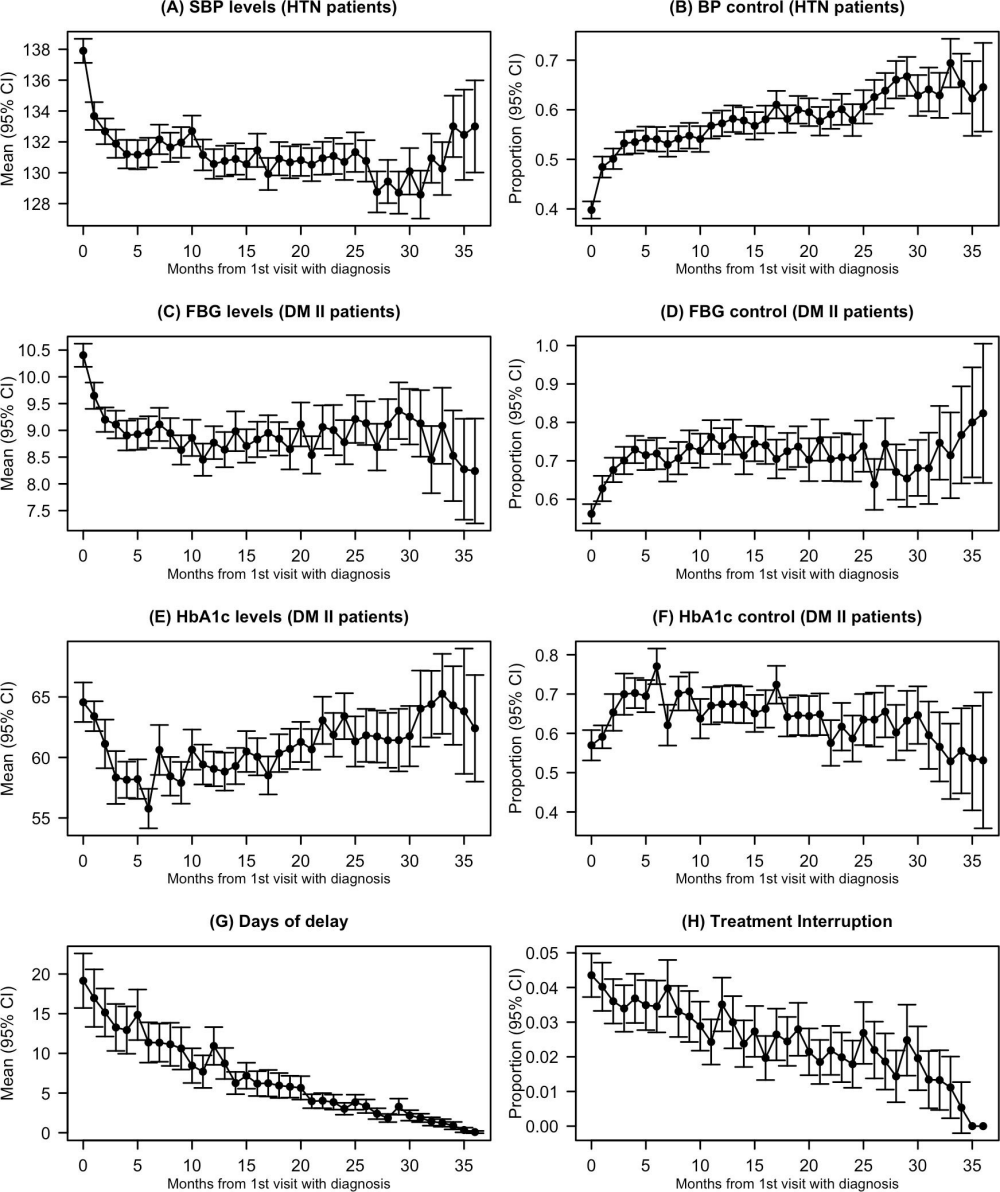
Patterns in control of clinical parameters and treatment interruptions from the patient perspective. (A) Mean systolic blood pressure (SBP; mm Hg) for all visits of patients with hypertension (HTN) per month since diagnosis. (B) Proportion of visits per month of patients with HTN at which BP control was achieved. (C) Mean fasting blood glucose (FBG) value (mmol/l) for all visits of patients with diabetes mellitus type II (DM II) per month since diagnosis. (D) Proportion of visits per month of patients with diabetes type II at which FBG target was achieved. (E) Mean glycosylated haemoglobin (HbA1c) value (mmol/mol) for all visits of patients with diabetes type II per month since diagnosis. (F) Proportion of visits per month of patients with diabetes type II at which HbA1c target was achieved. (G) Mean monthly days of delay (number of days after planned next visit date that next visit actually occurred) for all visits of patients with HTN and/or diabetes type II. (H) Proportion of visits per month of patients with HTN and/or diabetes type II at which a treatment interruption occurred (next visit was >31 days after next planned visit date). Each panel reports the average per visit value, using visits as the unit of analysis, for each month since diagnosis, for the reporting period December 2014 to December 2017 inclusive. ‘Diagnosis’ refers either to the month of enrolment for patients who had a known diagnosis on programme entry (90% of the cohort) or to month of new diagnosis for patients diagnosed at enrolment or at a subsequent visit. BP control was defined as BP < 140/90 mm Hg; control targets in patients with DM II were capillary FBG ≤ 180 mg/dl (10.1 mmol/l) or HbA1c < 8% (46 mmol/mol).

Fig 2 shows the monthly pattern in clinical control from the patient perspective, starting with the month of entry into the cohort, for those with established diagnoses, or the month when a new diagnosis was made.

#### Hypertension

The mean SBP in hypertensive patients decreased by 6.6 mm Hg within the first 6 months, from mean 137.9 mm Hg (95% CI 137.1 to 138.7) at entry/new diagnosis to 131.3 mm Hg (95% CI 130.3 to 132.3) after 6 months. The proportion achieving BP control improved from a baseline of 40% (95% CI 38% to 42%) to 54% (95% CI 52% to 57%) by month 6 after entry/new diagnosis.

#### Diabetes

Similarly, there was a marked improvement in FBG level by 1.43 mmol/l from a mean of 10.40 mmol/l (95% CI 10.19 to 10.62) at entry/new diagnosis to 8.97 mmol/l (95% CI 8.67 to 9.26) by 6 months; most of this improvement occurred within the first 3 months. From month 4 onwards, patients with DM II achieved FBG targets at over 70% of visits. The pattern in HbA1c (which reflects the previous 120 days of glycaemic control) was more variable. There was a rapid decline in the first 6 months, from a mean of 64.56 mmol/mol (95% CI 62.93 to 66.19) at the first visit to a mean of 55.77 mmol/mol (95% CI 54.14 to 57.41) after 6 months, but then a gentle slope upwards seemed to follow (Fig 2E). The percentage of visits where glycaemic control was achieved seemed to follow a similar pattern, with an increase in the first 6 months and then a levelling off or even a slight decrease in the following months (Fig 2F).

#### Treatment interruption

The mean number of days from the next planned appointment to the actual visit almost halved, from 19.2 (95% CI 15.7 to 22.6) in month 1 to 10.9 (95% CI 8.6 to 13.3) 12 months after entry/new diagnosis.

### Factors associated with clinical control and treatment interruption

In Tables 2 and 3 we report the multivariable linear and logistic regression analyses exploring factors associated with achieving BP/SBP, FBG, and HBA1c targets (using continuous and binary outcomes), with days of delay (continuous outcome), and with having at least 1 treatment interruption (binary outcome).

**Table 2:** Multivariable linear regression models to explore factors associated with achieving control of intermediate clinical outcomes and on days of delay (continuous outcomes).

Factor	Effect: Mean difference (95% CI)			
	BP control (mm Hg)	FBG control (mmol/l)	HBA1c control (mmol/mol)	Delay (days)
Previous treatment interruption	−0.02 (−1.21, 1.18)	−0.02 (−0.19, 0.16)	**1.12 (0.25, 2.00)**^a^	**−6.92 (−10.34, −3.50)**^c^
Male sex	0.75 (−0.57, 2.08)	**−0.42 (−0.65, −0.20)**^c^	−0.38 (−1.22, 0.47)	2.13 (−3.55, 7.81)
Age (per decade)	**2.58 (1.99, 3.17)**^c^	**−0.29 (−0.40, −0.19)**^c^	**−0.75 (−1.17, −0.34)**^c^	**−2.50 (−4.95, −0.04)**^a^
Syrian versus other nationality	0.96 (−0.53, 2.46)	0.07 (−0.18, 0.32)	−0.07 (−0.97, 0.84)	1.35 (−5.04, 7.74)
Per each year of programme	−0.76 (−1.84, 0.32)	−0.01 (−0.19, 0.17)	0.45 (−0.34, 1.25)	**−7.74 (−11.99, −3.50)**^c^
Per each year since diagnosis	−0.60 (−1.72, 0.51)	**−0.37 (−0.55, −0.19)**^c^	−0.10 (−0.89, 0.69)	**5.80 (1.47, 10.14)**^b^
Per additional NCD condition	**−0.56 (−1.09, −0.03)**^a^	−0.02 (−0.11, 0.06)	0.13 (−0.24, 0.51)	−1.05 (−2.92, 0.82)
Per additional medication	**−0.20 (−0.37, −0.02)**^a^	−0.01 (−0.04, 0.02)	−0.13 (−0.29, 0.03)	**0.90 (0.39, 1.41)**^c^
Hypertensive	—	−0.20 (−0.44, 0.04)	**−1.06 (−2.03, −0.08)**^a^	−0.44 (−6.02, 5.14)
Hypertensive, with controlled BP	—	**−0.22 (−0.33, −0.11)**^c^	**−1.00 (−1.68, −0.31)**^b^	1.36 (−0.66, 3.37)
Diabetic	**1.43 (0.01, 2.85)**^a^	—	—	**7.32 (1.61, 13.02)**^a^
Diabetic, with controlled FBG	**−2.20 (−3.15, −1.25)**^c^	—	—	**4.74 (2.31, 7.18)**^c^
February	−0.00 (−1.72, 1.72)	−0.18 (−0.42, 0.07)	0.06 (−1.58, 1.70)	−3.37 (−7.80, 1.05)
March	0.27 (−1.45, 1.98)	0.09 (−0.15, 0.33)	−0.03 (−1.53, 1.47)	2.84 (−1.59, 7.28)
April	−0.74 (−2.42, 0.93)	0.09 (−0.15, 0.33)	−0.39 (−2.02, 1.24)	−0.65 (−5.02, 3.71)
May	**−2.10 (−3.74, −0.47)**^a^	−0.05 (−0.28, 0.18)	**−3.81 (−5.34, −2.27)**^c^	−1.04 (−5.26, 3.19)
June	**−2.25 (−3.85, −0.64)**^b^	**0.58 (0.35, 0.81)**^c^	**−4.29 (−5.93, −2.64**^**)**^^c^	−0.10 (−4.27, 4.07)
July	**−2.37 (−4.06, −0.69)**^b^	0.14 (−0.10, 0.38)	**−4.14 (−5.71, −2.58)**^c^	−3.17 (−7.52, 1.19)
August	**−2.78 (−4.38, −1.18)**^c^	−0.17 (−0.40, 0.06)	**−2.53 (−4.05, −1.01)**^b^	0.11 (−4.04, 4.26)
September	**−1.73 (−3.43, −0.03)**^a^	−0.07 (−0.31, 0.17)	**−4.12 (−5.65, −2.58)**^c^	−2.05 (−6.44, 2.34)
October	−0.60 (−2.27, 1.07)	−0.04 (−0.27, 0.20)	−1.38 (−2.90, 0.14)	0.91 (−3.39, 5.21)
November	0.95 (−0.75, 2.66)	−0.17 (−0.41, 0.07)	−0.61 (−2.21, 0.99)	−1.79 (−6.18, 2.61)
December	**1.92 (0.27, 3.56)**^a^	0.04 (−0.19, 0.27)	−0.62 (−2.10, 0.85)	−0.88 (−5.14, 3.37)

Significant values in bold:

^a^*p-*value < 0.05,

^b^*p-*value < 0.01,

^c^*p-*value < 0.001.

BP control was defined as BP < 140/90 mm Hg; control targets in patients with diabetes mellitus type II were capillary FBG ≤ 180 mg/dl (10.1 mmol/l) or HbA1c < 8% (46 mmol/mol).

BP, blood pressure; FBG, fasting blood glucose; HbA1c, glycosylated haemoglobin; NCD, non-communicable disease

**Table 3:** Multivariable logistic regression models to explore factors associated with achieving control of intermediate clinical outcomes and with treatment interruption (binary outcomes).

Factor	Effect: Odds ratio (95% CI)			
	BP control	FBG control	HBA1c control	Treatment interruption
Previous treatment interruption	0.99 (0.84, 1.16)	0.96 (0.80, 1.16)	**0.86 (0.76, 0.97)**^a^	**1.55 (1.15, 2.08)**^b^
Male sex	0.88 (0.75, 1.02)	**1.53 (1.24, 1.90)**^c^	1.05 (0.94, 1.18)	1.06 (0.86, 1.31)
Age (per 10 years)	**0.83 (0.78, 0.89)**^c^	**1.27 (1.15, 1.41)**^c^	**1.10 (1.03, 1.16)**^b^	0.93 (0.85, 1.02)
Syrian versus other nationality	0.89 (0.75, 1.07)	0.99 (0.78, 1.25)	1.02 (0.90, 1.16)	0.95 (0.74, 1.22)
Per each year of programme	**1.16 (1.02, 1.33)**^a^	1.05 (0.88, 1.24)	1.00 (0.90, 1.12)	0.84 (0.67, 1.04)
Per each year since diagnosis	1.12 (0.98, 1.29)	**1.39 (1.17, 1.67)**^c^	0.97 (0.87, 1.09)	**0.75 (0.58, 0.97)**^a^
Per additional NCD condition	**1.15 (1.08, 1.23)**^c^	0.96 (0.88, 1.05)	1.01 (0.96, 1.07)	**0.80 (0.71, 0.89)**^c^
Per additional medication	**1.03 (1.01, 1.06)**^b^	0.98 (0.95, 1.01)	1.01 (0.98, 1.03)	1.03 (0.98, 1.08)
Hypertensive	—	**1.38 (1.10, 1.76)**^b^	**1.19 (1.04, 1.36)**^a^	1.06 (0.82, 1.38)
Hypertensive, with BP controlled	—	**1.28 (1.14, 1.44)**^c^	**1.18 (1.07, 1.30)**^b^	1.16 (0.94, 1.43)
Diabetic	0.90 (0.75, 1.07)	—	—	1.26 (0.93, 1.72)
Diabetic, with FBG controlled	**1.34 (1.17, 1.53)**^c^	—	—	1.21 (0.96, 1.54)
February	0.89 (0.70, 1.15)	1.23 (0.95, 1.60)	0.97 (0.78, 1.22)	0.92 (0.57, 1.50)
March	0.95 (0.74, 1.22)	0.94 (0.73, 1.22)	1.00 (0.81, 1.23)	0.94 (0.58, 1.52)
April	1.11 (0.87, 1.41)	1.03 (0.79, 1.33)	1.15 (0.92, 1.45)	1.01 (0.63, 1.61)
May	1.19 (0.94, 1.51)	1.12 (0.87, 1.44)	**1.78 (1.42, 2.21)**^c^	0.86 (0.54, 1.39)
June	1.17 (0.93, 1.47)	**0.63 (0.49, 0.80)**^c^	**1.79 (1.41, 2.27)**^c^	0.81 (0.50, 1.29)
July	1.20 (0.94, 1.53)	1.04 (0.80, 1.34)	**1.55 (1.24, 1.94)**^c^	0.90 (0.56, 1.47)
August	1.19 (0.94, 1.50)	**1.38 (1.07, 1.77)**^a^	**1.32 (1.07, 1.63)**^a^	1.09 (0.70, 1.70)
September	1.12 (0.87, 1.43)	1.14 (0.87, 1.48)	**1.55 (1.24, 1.92)**^c^	0.63 (0.37, 1.08)
October	1.10 (0.86, 1.40)	1.01 (0.78, 1.30)	1.01 (0.82, 1.25)	1.08 (0.68, 1.73)
November	0.81 (0.63, 1.03)	1.22 (0.94, 1.58)	1.12 (0.90, 1.40)	0.68 (0.40, 1.16)
December	**0.73 (0.58, 0.93)**^a^	1.04 (0.81, 1.34)	1.08 (0.88, 1.33)	0.68 (0.41, 1.14)

Significant values in bold:

^a^*p-*value < 0.05,

^b^*p-*value < 0.01,

^c^*p-*value < 0.001.

BP control was defined as BP < 140/90 mm Hg; control targets in patients with diabetes mellitus type II were capillary FBG ≤ 180 mg/dl (10.1 mmol/l) or HbA1c < 8% (46 mmol/mol).

BP, blood pressure; FBG, fasting blood glucose; HbA1c, glycosylated haemoglobin; NCD, non-communicable disease.

#### Hypertension

Among patients with HTN, mean SBP was higher by 2.58 mm Hg for each 10-year increase in age (95% CI 1.99, 3.17; *p* < 0.001), by 1.43 mm Hg with comorbid diabetes (95% CI 0.01, 2.85; *p* = 0.048), and by 1.92 mm Hg in the winter month of December (95% CI 0.27, 3.56; *p* = 0.022). However, visits with controlled diabetes and visits taking place in the warmer months (May to September) were associated with a reduction in SBP of approximately 2–3 mm Hg. Having additional NCD conditions and receiving additional medications were both associated with a small SBP reduction (less than 1 mm Hg), as shown in Table 2. Results for the logistic regression were similar, with target BP less likely to be reached during the winter months and more likely to be reached during the warmer months of the year.

#### Diabetes

Among patients with DM II, male sex, increasing age, years since diagnosis, and having controlled BP were all strongly associated with improved FBG control. There was a notable deterioration in the month of June, leading to a mean increase of 0.58 mmol/l (95% CI 0.35, 0.81; *p* < 0.001). Logistic regression results showed that in addition to the above factors, a diagnosis of comorbid hypertension increased the odds of achieving target FBG. Improved mean HbA1c was associated with increasing age, a comorbid diagnosis of HTN (and, if hypertensive, with having controlled BP), and warmer months (May to September). Having a previous treatment interruption slightly increased mean HbA1c by 1.12 mmol/mol (95% CI 0.25, 2.00 *p* < 0.05). The same factors influenced whether the HBA1c target was reached.

#### Treatment interruption

The mean number of days the next visit took place after the planned appointment date was significantly decreased by having had a previous treatment interruption, with increasing age, and with maturation of the programme. Risk of delayed attendance for the next appointment increased with time since enrolment/diagnosis, with each additional NCD medication prescribed, with having a diagnosis of diabetes, and with having FBG at target at the index appointment. It was not affected by calendar month. The risk of treatment interruption decreased with time since enrolment/diagnosis and with each additional NCD diagnosis. It was increased by having a previous interruption (Table 3).

## Discussion

To our knowledge, this is the first study describing clinical outcomes in a primary-level NCD programme for Syrian refugees and the vulnerable host population in Jordan. Overall, we found that intermediate clinical outcomes improved, and the risk of treatment interruption decreased, with programme maturation. From a programmatic perspective, the greatest gains in SBP and FBG control occurred in the first 6 months of the programme. From a patient perspective, clinical parameters also improved in the initial months after entry into the programme (or after diagnosis).

We found a marked seasonal variation in SBP control, with an increase in the mean of up to 5 mm Hg during the colder winter months, which was confirmed by regression analyses. An inverse relationship between ambient temperature and BP (with higher BP reported during colder weather and vice versa) has been identified previously, but, to the best of our knowledge, this had not been reported in humanitarian settings [28–30]. The opposite was true for diabetes control. The regression analyses showed the month of June was associated with a marked deterioration in FBG control. There were no known programmatic or service use changes, such as an increase in new patient intake (S2 Fig), to explain this, and we postulate that it may coincide with fasting during the holy month of Ramadan followed by the 3-day Eid ul-Fitr festival [17].

From the patient perspective (the cohort analysis), the marked improvement in SBP control within the first 6 months among patients with HTN (mean drop 6.6 mm Hg) is similar to that seen in other studies [23,31]. A clear improvement within the first 6 months was also seen in diabetic patients. Thereafter, time in programme or programme duration did not appear to affect the odds of achieving glycaemic control. We note, however, that the proportion achieving the HbA1c target by month 12 (67%) was lower than the proportion attaining FBG control (74%), potentially reflecting patients’ improved treatment adherence in the days around their clinic appointments, as has been shown in other settings [32]. However, other potential explanations concern the variable relationship between mean plasma glucose and HbA1c and the individual variability in haemoglobin glycation rates, due to different erythrocyte longevity and genetic factors, which may explain the apparent reduced HbA1c sensitivity in Arab populations [27,33].

Increasing age was associated with lower odds of achieving BP control. While BP rises with age, and elevated BP generally requires additional classes and higher doses of antihypertensive drugs over time, further work is needed to determine whether patients with uncontrolled HTN had resistant HTN or whether they were undertreated. Conversely, increasing age and having comorbid HTN were associated with better diabetes control. This may reflect the fact that older patients with multi-morbidity received greater clinical attention or were more adherent to treatment. These differences may have implications for programme planning as the factors to consider in bringing about improvements in one clinical parameter may vary from those for another.

Syrians made up approximately two-thirds of the cohort, and the remainder were mainly Jordanian, reflecting Jordanian policy that access to humanitarian programmes should be extended to the host population. In our analysis, nationality did not impact the odds of reaching clinical targets, despite the Syrian cohort being more vulnerable, less educated, and generally poorer, with a decreased capacity to access care than their Jordanian counterparts, as highlighted by other authors [10,34]. Having a previous treatment interruption was the main risk factor for a further episode of treatment interruption, as might be expected, while the number of days of delayed attendance decreased with programme maturation. It may be that patients retained in the cohort were potentially more adherent than those who stopped attending, but this finding may also reflect the fact that clinic staff strongly emphasised adherence to appointments, achieving a 90% attendance rate by 2017 (MSF data). Patients with FBG at target were more likely to delay attending their next planned appointment or to have a treatment interruption yet maintained FBG control, perhaps highlighting that patients with controlled diabetes could be given greater intervals between appointments.

The number of published studies that include outcomes on the clinical effectiveness of NCD care models in humanitarian settings is growing from a low baseline [13]. UNRWA published several cohort studies assessing clinical outcomes in cumulative and quarterly cohorts of new admissions using an electronic medical record. They reported similar proportions achieving SBP < 140/90 mm Hg (76%) and glycaemic control (between 50% and 78%, using a target of 2-hour postprandial glucose ≤ 180 mg/dl [10.1 mmol/l]), with improved testing rates over time but rising rates of loss to follow-up and complications [21,22,35,36]. Several studies of MSF NCD cohorts using routinely collected clinical data have now been published utilising different service models, lengths of follow-up, treatment targets, and statistical analyses [23,31,37,38]. In integrated NCD/HIV programmes in Cambodia and in Kenya, 49.3% (*n =* unknown) of non-diabetic hypertensive patients and 50% (*n =* 466) of HIV-negative patients achieved SBP control (<140 mm Hg), while amongst Syrian refugees in Lebanon, 49% (*n =* 75) of non-diabetic hypertensive patients achieved BP targets (<140/90 mm Hg). In the Kenyan and Cambodia cohorts, less than a quarter (19% [*n =* 26] and 24% [*n =* 51]) reached the target HBA1c of <7%, while, in Lebanon, 61% (*n =* 40) reached the more conservative target of <8%, similar to our findings.

The HbA1c and FBG target levels used in this analysis, drawn from the MSF NCD guidelines, were less strict than international norms, which is reflected in the comparatively high rates of control. If we reanalyse using a target HbA1c of <7% rather than <8%, the proportion of visits meeting the target 6 months post-enrolment drops from 77% (95% CI 73%–82%) to 53% (95% CI 47%–58%). Since many patients in this study would have met the stricter treatment targets suggested by international bodies such as the American Diabetes Association (HbA1c 6.5%–8%), it may be reasonable to introduce tighter, individualised treatment targets for many patients in this context, as suggested in similar humanitarian settings [23,39].

We also note that the high levels of clinical control seen in this study were achieved in the setting of a complex, facility-based, multidisciplinary programme and may reflect MSF’s substantial resources and programmatic experience. In addition, consultations, medications, and laboratory investigations were provided free of charge to patients, as is MSF’s standard practice, which removed the main cost barrier to NCD care reported by Syrian refugees in Jordan [9,10,34]. However, this service was limited in coverage (reaching only 23% of adults with self-reported NCDs in Irbid governorate) and in scope, treating a limited number of medical conditions [9]. Multiple studies have highlighted the complex, fragmented, and inadequate NCD care available to Syrian refugees in Jordan and neighbouring host countries [7,10,40]. The high cost of NCD care remains the principle barrier to the MOH, UNCHR, and other actors providing more comprehensive NCD services, while patients often face unaffordable co-payments and transport costs to access existing services [7,10]. Qualitative data from our RE-AIM evaluation confirmed that Syrian refugees in Jordan attempt to meet their perceived NCD care needs by attending a mix of providers (public, NGO, and private facilities or pharmacies) and by carefully balancing costs and household income [41]. When they are financially stretched, they cope by rationing, borrowing, or begging, often depending on the generosity of the Syrian and Jordanian communities [10]. Solutions to increase the coverage of high-quality, standardised, and cost-effective primary-level NCD care and to improve access to essential secondary and tertiary investigations and interventions are urgently needed.

### Strengths and limitations

A key strength of this paper is that changes in clinical patterns were explored both using calendar time (programmatic perspective), which may be useful for programme managers, and using patients’ time in the programme (closed cohort approach), which is useful for clinicians. The former allowed us to identify and adjust for seasonality in BP control, which a cross-sectional approach at a single time point may have missed. Additionally, we maximised use of the rich, continuous routine data collected by the programme at every patient visit, given that all enrolled adults with HTN and DM II contributed data to the analysis, regardless of whether they had periods of treatment interruption or were lost to follow-up. An additional important strength is that we conducted a multivariable analysis using random effect models that explored the association of patient, programme, and contextual factors with each of the outcomes.

The limitations include that this was a retrospective implementation study of a complex intervention in an unstable humanitarian setting and, thus, subject to the significant challenges inherent in performing evaluations in such contexts. These include using routine clinical and programmatic data, which may have missing variables and be of limited quality. However, the dynamic nature of the context and interventions preclude implementing more traditional experimental designs (such as randomised controlled trials). Our study design did not allow us to establish the causal mechanisms responsible for the reported outcomes or to attribute effectiveness to individual programme components, but we explored associations that could be analysed in future studies [42,43]. We did not focus on other NCD comorbidities, such as chronic respiratory disease and CVD, which are potentially more complex to diagnose and treat at the primary care level and may have achieved less favourable outcomes [44]. We used intermediate clinical outcomes to analyse programme quality since following up hard outcomes, such as mortality and complication rates, requires time. Monitoring these hard outcomes is more complex in humanitarian settings due to high attrition rates, mobile populations, and poor communication links with secondary care, where many NCD-related deaths are likely to occur. Finally, our outcome results were compared to targets that had been adapted to the humanitarian context and may not represent ideal targets for best clinical outcomes.

### Implications for practice, policy, and research

Since a previous treatment interruption was the main risk factor for delayed appointment attendance and future treatment interruptions, MSF could consider specific interventions and strategies to support access and continuity of care for this group, ideally developed in consultation with them. For example, these may include person-centred options such as flexible opening hours or decentralisation of certain aspects of care to the community level through use of community adherence groups or technology to promote self-monitoring (eHealth). Limited evidence shows that there is no benefit to reviewing stable patients more often than 6-monthly (depending on severity, comorbidities, etc.), so service use could potentially be rationalised by extending appointment intervals for those with good disease control [45].

MSF may also consider revising their BP and HbA1c targets in the Middle East population to stricter, individualised treatment targets, in line with international norms, given the high rates of control. The service may need greater focus on achieving SBP control in older patients, as clinically appropriate. This may involve both exploring whether prescriber fatigue, patient non-adherence, and/or resistant HTN are contributing to the poorer BP control in this group and introducing individualised treatment targets. More lenient treatment targets may also need to be considered during winter months. Diabetic patients could potentially benefit from additional support and advice during Ramadan.

Further research is needed to improve monitoring and evaluation tools to (1) utilise the continuous routine data being collected by some NCD programmes in humanitarian settings, (2) determine the important components for quality assessment of these programmes, and (3) explore the effectiveness of the individual components of NCD programmes and explore causal mechanisms, nesting randomised controlled studies within programmes (for more narrow or specific questions) and using novel methodological approaches such as causal inference frameworks appropriate to the evaluation of complex interventions.

Lessons learned around the implementation of this programme may be useful for the Jordanian MOH system as it continues to strengthen primary-level NCD care. We note the increasing role played by MSF nurses in health education, monitoring, and, more generally, management of stable patients with NCDs. Given the pressure on the Jordanian health system and the pull of the private sector and Gulf countries on Jordanian medical practitioners, empowering nurses to play a greater role in the provision of primary-level chronic disease care may be an option for policy makers, regulators, and training bodies to consider. We also note that the positive results presented here were achieved in a relatively complex and costly programme [46]. There may be scope to design and test simplified, more cost-efficient, person-centred programmes that include features such as fixed dose combination therapy, reduced frequency of clinic contact, decentralisation of care to the community, empowerment of patients to self-care and to act as peer supporters, and increased use of technology in this tech-savvy population [47]. Any such modifications to the care model would require careful evaluation of their feasibility and acceptability to policy makers, practitioners, and patients.

### Conclusion

In conclusion, we have added to the existing evidence base around the NCD disease burden and barriers to accessing care among Syrian refugees in Jordan, by describing the content and quality of a specific NCD programme designed to meet these patients’ needs. This study shows that good treatment outcomes and reasonable rates of treatment interruption can be achieved in a multidisciplinary, primary-level NCD programme in north Jordan. We suggest that monitoring and evaluation of NCD programmes could be further improved by building on the analysis we have presented here. We also suggest that this comprehensive model of care could be adapted to make it more person-centred, more cost-efficient, and more easily replicated in Jordan and similar contexts in the Middle East.

## Supporting information

S1 Fig MSF Irbid NCD programme timeline. (XLSX)

S2 Fig Monthly new and follow-up appointments by nationality at the MSF NCD clinic in Irbid, Jordan (January 2015 to December 2017). (DOCX)

S1 Protocol MSF London School of Hygiene & Tropical Medicine Irbid, Jordan, NCD evaluation protocol. (DOCX)

S1 STROBE Checklist (DOC)

## Abbreviations

BP: blood pressure
CVD: cardiovascular disease
DM II: diabetes mellitus type II
FBG: fasting blood glucose
HbA1c: glycosylated haemoglobin
HTN: hypertension
MOH: Ministry of Health
MSF: Médecins Sans Frontières
NCD: non-communicable disease
NGO: non-governmental organisation
SBP: systolic blood pressure
UNRWA: United Nations Relief and Works Agency for Palestinian Refugees
