# Correction: Clinical outcomes in a primary-level non-communicable disease programme for Syrian refugees and the host population in Jordan: A cohort analysis using routine data

**DOI:** 10.1371/journal.pmed.1004373

**Published:** 2024-03-22

**Authors:** Éimhín Ansbro, Tobias Homan, David Prieto Merino, Kiran Jobanputra, Jamil Qasem, Shoaib Muhammad, Taissir Fardous, Pablo Perel

There are errors in the Funding and Competing Interests statements. The correct statements are:

**Funding:** Médecins Sans Frontières (MSF) provided support for this study in the form of salaries for TH, KJ, JQ, and SM. EA, DPM, and PP received funding from Médecins sans Frontières UK as part of a research fellowship awarded to EA. The specific roles of these authors are articulated in the ‘author contributions’ section. MSF played a role in study design, data collection, analysis, decision to publish and preparation of the manuscript.

**Competing Interests:** The authors have read the journal’s policy and have the following competing interests to declare: Médecins Sans Frontières (MSF) provided support in the form of salaries for TH, KJ, JQ, and SM. This does not alter our adherence to *PLOS Medicine* policies on sharing data and materials. There are no patents, products in development, or marketed products associated with this research.
