# Supplementary material for: Clinical outcomes in a primary-level non-communicable disease programme for Syrian refugees and the host population in Jordan: A cohort analysis using routine data
Source: PLoS Med. 2021 Jan 11;18(1):e1003279. doi: 10.1371/journal.pmed.1003279 (PMC7799772; doi:10.1371/journal.pmed.1003279)
Supplement: S2 Fig — (DOCX) [file pmed.1003279.s002.docx]

**
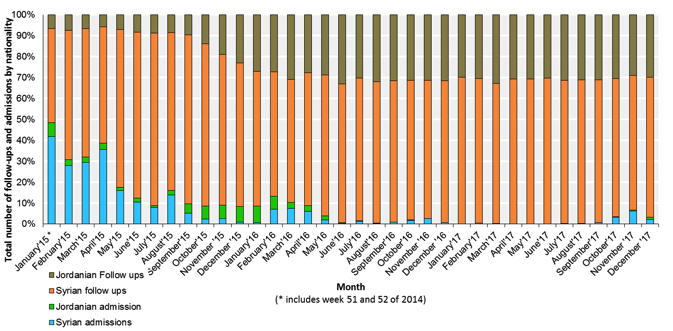
S3. Figure. Monthly new and follow up appointments by nationality at MSF NCD Clinic in Irbid, Jordan (January 2015 to December 2017)**
