# Supplementary material for: Clinical outcomes in a primary-level non-communicable disease programme for Syrian refugees and the host population in Jordan: A cohort analysis using routine data
Source: PLoS Med. 2021 Jan 11;18(1):e1003279. doi: 10.1371/journal.pmed.1003279 (PMC7799772; doi:10.1371/journal.pmed.1003279)
Supplement: S1 Protocol — (DOCX) [file pmed.1003279.s003.docx]

- **Médecins sans Frontières / London School of Hygiene & Tropical Medicine**

**Evaluation of a primary care-based NCD service in Irbid, Jordan, 2017**

Research Study Protocol

Final

# Key data

| Version | Final (v.1.5) |
| --- | --- |
| Study design | Mixed methods (cohort, qualitative) |
| Study period | February 2017 to February 2018 |
| Study sites | 2 MSF NCD clinics including Mental Health and Home Visit service, Irbid, Jordan |
| Principal investigator | Dr Éimhín Ansbro, Researcher, LSHTM ([Eimhin.Ansbro@lshtm.ac.uk](mailto:Eimhin.Ansbro@lshtm.ac.uk)) |
| Co-investigators | Dr Tobias Homan, Epidemiologist, MSF (OCA) Jordan  Dr Kiran Jobanputra, NCD Advisor, MSF (OCA) ([kiran.jobanputra@london.msf.org](mailto:kiran.jobanputra@london.msf.org))  Dr Sarah N.M.A.G. Ellithy (Humanitarian Liaison Officer MSF OCA)  Hashim Taani (Humanitarian Liaison Officer MSF OCA)  Dr Mohammed Shoaib, Medical Coordinator, MSF (OCA) Jordan  Dr Mohammad Tarawneh, Primary Care Director, Ministry of Health of,Jordan  Dr.Majed Assad, NCD Directorate, Ministry of Health of Jordan  Dr Taissir Fardous, Health Economy Directorate. Ministry of Health of Jordan  Dr Myassar Zindah, Cardiovascular Disease Dept., Ministry of Health of Jordan  Dr Bayard Roberts, Research Advisor, LSHTM ([Bayard.Roberts@lshtm.ac.uk](mailto:Bayard.Roberts@lshtm.ac.uk))  Dr Pablo Perel, Research Advisor, LSHTM ([Pablo.Perel@lshtm.ac.uk](mailto:Pablo.Perel@lshtm.ac.uk))  Dr David Prieto, Statistician, LSHTM ([David.Prieto@lshtm.ac.uk](mailto:David.Prieto@lshtm.ac.uk))  Dr Zia Sadique, Economics Advisor, LSHTM ([zia.sadique@lshtm.ac.uk](mailto:zia.sadique@lshtm.ac.uk)) |
| Study sponsor | MSF-UK (Manson Unit) |

# List of acronyms

AMR Arab Medical Relief

BMI Body Mass Index

BP Blood Pressure

COPD Chronic Obstructive Pulmonary Disease

CVD Cardiovascular Disease

DM Diabetes Mellitus

FPG Fasting Plasma Glucose

Hb Haemoglobin

HbA1c Glycosylated Haemoglobin

HE Health Education

HLO Humanitarian Liaison Officer

HTN Hypertension

LFT Liver Function Tests

MH Mental Health

MHPSS Mental Health and Psychosocial Support

MoH Ministry of Health

MSF-OCA Médecins sans Frontières – Operational Centre Amsterdam

MSF-OCBA Médecins sans Frontières – Operational Centre Barcelona

MSF-UK Médecins sans Frontières – United Kingdom Office

NCD Noncommunicable Disease

NGO Nongovernmental Organisation

RE-AIM Reach, Effectiveness, Adoption, Implementation, Maintenance

UNHCR United Nations High Commissioner for Human Rights

WHO World Health Organization

# Executive Summary

**Background:** Non-communicable diseases (NCDs), particularly diabetes and cardiovascular disease, are some of the leading causes of mortality and morbidity among Syrian refugees in Jordan. Since the start of the Syrian crisis in 2011 the health system in northern Jordan has been overwhelmed by the burden of chronic disease amongst Syrian refugees, such that it has been obliged to scale up primary level care for NCDs. Historically, this care was provided at secondary or tertiary level in Jordan. In December 2014, MSF commenced an NCD service at two primary care centres in Irbid using a multi-disciplinary primary care model with task shifting, context-adapted clinical guidelines, adapted patient counselling and support materials and medications from the World Health Organization (WHO) Essential Medicines list. The programme focuses on the NCDs and NCD risk factors responsible for the greatest mortality in pre-war Syria: cardiovascular disease, including hypertension, diabetes types I and II, asthma and chronic obstructive pulmonary disease (COPD). This programme represents an opportunity to evaluate and refine a model of NCD care delivered in a humanitarian setting in order to support its application in comparable settings.

**Overall aim:** To evaluate a primary care based model of NCD care in Irbid, Jordan in order to refine the model and to generate evidence on its feasibility, acceptability and effectiveness with a view to translating a similar model to comparable humanitarian settings.

**The specific objectives** are to examine the**:**

- *Reach* (coverage) of the NCD service and its components to the intended target population.
- *Effectiveness* of the NCD service. We defined “effectiveness” for this evaluation as quality of care. This will be examined by identifying trends in clinical outcomes and quality of care indicators; exploring perceived benefits, unintended consequences, behavioural outcomes and evaluating economic outcomes.
- *Adoption / acceptance* of the NCD service and its components (including medication adherence) by the organisation, setting, staff and patients, and consequent changes to behaviour and practice.
- *Implementation* of the NCD service and its components including fidelity in terms of consistency of applying the defined guidelines and processes; guideline usability, adaptation of structures, processes and tools; and costs.
- *Maintenance* of the NCD service and its components in patients, programme and organisation over time.

**Methods:**

*Design*: A mixed-methods design will be used, based upon the RE-AIM framework (see <http://www.re-aim.hnfe.vt.edu/>). Objective 1 (Reach) will refer to a pre-existing dataset from a random cross-sectional population-level household survey previously conducted by MSF for separate purposes; routine cohort data from two MSF clinics in Irbid; and qualitative data from focus groups, semi-structured and key informant interviews. Objective 2 (Effectiveness) will use a cohort study design of chronic disease patients at the MSF-run clinics in Irbid. It will use routine clinic data to explore trends in clinical outcomes and other quality of care indicators, which occurred during MSF’s implementation of this NCD care model in Irbid. It will also include descriptive analysis of total and unit programmatic costs. Objective 3 (Adoption/acceptance) will use qualitative data to explore issues around adoption and acceptance of the model components through participant observation, focus group discussions and semi-structured interviews with patients, staff and key stakeholders. A small exploratory study to develop and test a short survey of self-reported medication adherence and beliefs will also be undertaken. Objective 4 (Implementation) will use routine cohort data, clinical audit, participant observation, routine costing data and qualitative data (as described above). Objective 5 (Maintenance) uses routine cohort data, qualitative data (as described above) and data on medical supplies, costs and staff time estimates.

*Study participants:* The pre-existing cross-sectional health service access survey included Syrian refugees households living outside of camp settings in Irbid governorate, selected using a two-stage cluster design. For the routine cohort data, all patients six years and older with confirmed NCDs attending the two MSF clinics in Irbid more than once, will be included (using patient records from January 2015 to December 2016). For the qualitative research, respondents will be purposively selected patients and/or health workers, past and present, from the two MSF clinics in Irbid. Key stakeholder interviews will include staff from MSF and other NGOs, Jordanian MoH staff and community leaders. For the medication adherence survey, a random sample of 300 consenting service users attending the MSF clinic will be selected.

*Data collection*: Household survey data were previously collected by trained interviewers using an electronic tablet-based survey tool and uploaded to a password-protected server. Trained data clerks will collect routine cohort data from routine patient records and will input this data daily into a predesigned database. Clinical audit data will be collected on a paper-based checklist from routine patient records and entered into a purpose-designed Excel spreadsheet. Cost data will be obtained from accounting records, supply orders and staff observation. Qualitative data will be collected by trained interviewers using topic guides. The adherence survey will utilise adapted pre-existing self-report medication adherence and beliefs measures with data entered into a purpose-designed Excel spreadsheet.

*Data analysis:* Secondary analysis of the pre-existing household survey data will use descriptive methods and multivariate regression modelling. For the cohort study, descriptive analysis and multivariate logistic regression analysis will be used to explore the clinical outcome trends, complication rates, quality of care and guideline adherence that occurred during implementation of this model. Data from the clinical audit and from the adherence survey will be analysed using descriptive statistics. Costing analysis will describe total and unit costs and will compare cost levels and trends across different time periods as new programmatic elements were added. The qualitative research will use thematic analysis. Qualitative and quantitative data from the various data sources will be triangulated.

**Ethical considerations:** This study protocol will be submitted to the MSF Ethics Review Board and LSHTM Ethics Committee for ethics clearance. Written authorisation to implement the study will also be obtained from the MoH of Jordan.

**Dissemination:** Ongoing data from the study will be shared with local, national (including the Jordanian MoH) and international stakeholders every 6 months in order to inform and improve the delivery of NCD care by MSF and other public health agencies in the study area. Outputs will include reports and peer-reviewed academic publications and presentations at key conferences and workshops.

Table of Contents

Key data 2

List of acronyms 3

Executive Summary 4

1. Background 12

1.1 MSF Model of NCD Care in Irbid 13

1.1.1 NCD Service 14

1.1.2 Patient circuit 15

1.1.3 MSF NCD Clinical Guidelines 18

1.1.4 Evolution of model of care 19

1.1.5 Programmatic Changes over Time 19

1.1.6 Existing data sources, records and assessment processes 20

2. Study rationale, aim and objectives 22

3. Methods 23

3.1 Description of the study sites 23

3.2 Overall study design 30

3.3 Study population 30

3.4 Cohort study method 31

3.4.1 Cohort study area and study population 32

3.4.2 Cohort study main outcome measures 33

3.4.3 Cohort data sources 34

3.4.4 Cohort data management 35

3.4.5 Cohort data analysis 35

3.4.6 Clinical audit 36

3.4.7 Costing analysis 36

3.5 Self-administered medication adherence questionnaire 37

3.5.1 Adherence questionnaire methodology 37

3.5.2 Adherence questionnaire population 37

3.5.3 Adherence questionnaire eligibility criteria, recruitment and sampling 38

3.5.4 Adherence questionnaire data tools 38

3.5.5 Adherence questionnaire data management 39

3.5.6 Adherence questionnaire data analysis 39

3.6 Cross-sectional household survey data analysis 39

3.6.1 Household survey population 40

3.6.2 Household survey eligibility criteria and sampling 40

3.6.3 Household survey data collection and management 41

3.6.4 Household survey data analysis 41

3.7 Qualitative research 41

3.7.1 Qualitative methods 42

3.7.2 Qualitative research study population and sampling 43

3.7.3 Qualitative data collection 44

3.7.4 Quality assurance 46

3.7.5 Qualitative data analysis 47

4. Ethical considerations 49

4.1 Social value 49

4.2 Potential risks from the study 49

4.3 Respect for and protection of recruited participants and study communities 50

4.4 Informed consent 50

4.5 Safety considerations 52

4.6 Data management and protection 53

4.7 Confidentiality 53

4.8 Independent review 53

5. Study implementation 54

5.1 Collaborative partnership 54

5.2 Timeline 54

5.3 Dissemination plan 54

5.4 Financial resources 55

6 Limitations 55

7 References 57

Annex 1: Consent Forms 61

Annex 1a: Consent statement for patients of MSF NCD service for the qualitative research (focus group discussions) 61

Annex 1b: Consent statement for patients of MSF NCD service for the qualitative research (semi-structured interviews) 67

Annex 1c: Consent statement for patients of MSF NCD service for the medication adherence questionnaire 72

Annex 1d. Consent statement for health staff for the qualitative research 76

Annex 1e. Consent statement for key stakeholders for the qualitative research 79

Annex 1f. Consent statement for observations of patient group counselling and clinical consultations 82

Annex 2: Data collection forms 85

Annex 2a: Topic guide – Focus group discussions with NCD patients 85

Annex 2b: Topic guide – Semi-structured interviews with NCD patients 87

Annex 2c: Topic guide – Semi-structured interviews with NCD health care providers and staff 89

Annex 2d: Topic guide – Semi-structured interviews with key stakeholders 91

Annex 2e: Clinical Audit Sample Checklist 93

Annex 2f: Patient Self-report Medication Adherence and Beliefs Questionnaire 94

# 1. Background

NCDs have been responsible for the majority of deaths worldwide for more than three decades, causing 68% of the 56 million global deaths in 2012 ^1^. NCDs accounted for 77% of mortality in pre-conflict Syria, with cardiovascular disease the leading cause of death ^2^. Data from the UNHCR and other actors has confirmed the high burden of NCDs amongst Syrian refugees in Jordan ^3,4^. As in other settings, the majority of these refugees live amongst the local community and traditional camp-based care-provision has had to be adapted to support refugees living in informal urban settings ^4–6^. In response to the overwhelming burden of chronic disease amongst urban-dwelling Syrian refugees, the health system in north Jordan has been obliged to scale up primary-level NCD care. Historically, this care was provided at secondary or tertiary level in Jordan. Médecins sans Frontières (MSF), a humanitarian emergency medical organisation, has supported the Jordanian health system in providing NCD care to the Syrian refugee and vulnerable Jordanian population in Irbid, Jordan’s second largest city, since 2014.

In any setting, patients with NCDs tend to be older, have multiple, co-morbid chronic conditions and are prescribed several medications. They require long-term, continuous medical care and psychosocial support rather than the acute, episodic care that usually characterises healthcare in humanitarian settings ^7–10^. In addition, chronic medical conditions and mental illness or distress often co-exist; depression is more common in people with chronic disease compared to the general population and can worsen associated health outcomes ^11–13^. We can assume that NCD and mental health co-morbidities are more frequent still amongst refugees fleeing a protracted civil war.

The attention of the global health community has recently shifted to include chronic NCDs, focusing on four core NCDs (cardio-vascular diseases, diabetes, chronic respiratory diseases and cancer) with shared characteristics: they are increasingly common due to globalisation; they are responsible for an epidemiological shift in low- and middle-income countries (LMICs); they share common, behaviourally-modifiable risk factors; and they are amenable to testing and treatment at primary care level. To improve NCD outcomes in LMICs, WHO and others advocate strengthening person-centred, primary-level NCD care, which involves use of: evidence-based treatment protocols (which involve global cardiovascular risk management, lifestyle modification and supported self-care), a core set of generic medications and basic technologies ^14,15^.

While a wealth of scientific evidence on cost-effective, primary care-based clinical management of NCDs exists, little evidence is available to guide the delivery of such interventions in LMICs. The research evidence base and the MSF institutional experience to support NCD programming in humanitarian settings are still more limited ^16,17^. Thus, this programme, described in detail below, was initiated to meet an identified healthcare gap for Syrian refugees, and has evolved in response to the clinical, technical and programmatic needs that have emerged over time. This process has resulted in a multidisciplinary care model, which includes specific humanitarian and mental health and psychosocial support (MHPSS). This evaluation provides an opportunity to refine a model of NCD care delivered in a humanitarian setting in order to improve patient care and to support the application of similar programmes in comparable settings.

## 1.1 MSF Model of NCD Care in Irbid

Using a primary care model, described in detail below, the NCD service is based on a multidisciplinary approach with task shifting; context-adapted clinical guidelines and tools; adapted health education and mental health and psychosocial support (MHPSS); and medications included in the World Health Organization (WHO) Essential Medicines list.


### 1.1.1 NCD Service

The MSF NCD programme’s target population comprises urban-based Syrian refugees, both United Nations High Commissioner for Human Rights (UNHCR) registered and unregistered, and vulnerable Jordanians (those not entitled to or otherwise unable to access subsidised Ministry of Health services). At present, MSF is the only provider of free primary-level, specialised NCD care to Syrian refugees in Irbid governorate; other non-governmental organisations (NGOs) require co-payments for similar services. In December 2014, MSF commenced an NCD programme in Irbid within an MoH primary care centre at Ibn-Sena. A second clinic was opened at Ibn-Rushd in April 2015 alongside a private medical organisation. In 2016, a separate, similar NCD programme was opened by MSF-Operational Centre Barcelona (MSF-OCBA) in Ramtha, a border town in Irbid governorate, but it is not included in this cohort evaluation.

To date (November 2016), over 3500 patients have been enrolled in the Irbid programme. The service initially consisted of triage by nurses, routine medical review by doctors, health education provided by health promoters at each clinical contact, and provision of free medications dispensed from the on-site pharmacy by trained pharmacists. Specific programme components were added over time in response to emerging patient, programmatic and contextual needs: a mental health and psychosocial support (MHPSS) service for patients with specific indications; a humanitarian liaison officer who links patients to other humanitarian services; and a home visit service for frail or immobile patients.

The MSF NCD programme focuses on the four most common NCDs or NCD risk factors, which were responsible for the highest mortality amongst Syrians before the current conflict^2^: cardiovascular disease (CVD), including: hypertension (HTN), myocardial infarction, angina pectoris, stroke, transient ischaemic disease, peripheral vascular disease; diabetes mellitus types I and II (DM); asthma and chronic obstructive pulmonary disease (COPD); and co-morbid mental health issues which may impact on NCD care, such as bereavement, anxiety and depression. Patients with these named NCDs or risk factors are admitted to the programme and the programme guidelines, treatment protocols and data collection tools focus on these diseases. Other NCDs are managed in different ways as follows:

1. Patients who have an NCD other than those named above are not admitted to the programme but are referred to the MoH or other service providers where available e.g. isolated hypothyroidism is referred to the International Rescue Committee clinic.

2. Co-morbid NCDs in addition to those named above, which may be treated at primary care level with medications included in the MSF Essential Drug List, are managed within the programme e.g. providing analgesia for osteoarthritis pain or anti-acid medications for gastritis.

3. NCDs that are not managed by the programme currently e.g. cancer, or complications of the named NCDs requiring secondary or tertiary care input, are referred to MoH services or to other NGOs where available e.g. retinal screening for diabetic patients is provided by the Qatari Red Crescent hospital in Irbid.

### 1.1.2 Patient circuit

A basic primary-level NCD service was initially introduced at each clinic. The typical patient circuit is as follows: patients are directed at reception to the MSF NCD service if they have both a medical indication (suspected or confirmed target NCD or risk factor) and a social indication (Syrian refugee or vulnerable Jordanian). All other cases are directed to the standard MoH (or private) primary care service.

Patients are initially triaged by nurses who record socio-demographic data; measure height, weight, waist circumference, blood pressure, heart rate, capillary blood sugar level, temperature and oxygen saturations; and calculate body mass index. Doctors provide medical consultations according to setting-specific protocols. These were initially based on Jordanian and international guidelines and were later adapted in line with MSF Operational Centre Amsterdam (MSF-OCA) NCD guidelines (see section 1.1.3 for more detail).

At first visits, doctors record a complete medical, medication and family medical history and perform a clinical examination for each patient. Lifestyle CVD risk factors (smoking status, alcohol intake, exercise levels) are recorded; global cardiovascular risk score is calculated; acute complications are identified and treated; long-term medications are prescribed for symptom management and secondary prevention of complications; patients are referred for laboratory testing; and a follow-up interval is determined. Patients with specific needs are referred internally (see below) or externally to the Jordanian public health services or to other NGOs, as described earlier.

Follow-up visits involve review of patients’ symptoms and disease control, vital signs, laboratory results; determination and recording of new diagnoses; adjustment and / or initiation of medications; and referral for further laboratory tests or to other health providers as required. Doctors also cover health education at each consultation and refer all patients to the programme health promoters.

Health promoters provide individually tailored health education at each clinical contact. Topics covered include: education about diagnosis and disease, treatment and adherence support, patient concerns and expectations, and lifestyle changes. Motivational interviewing techniques are used to support patients to make lifestyle modifications related to diet, exercise levels and smoking. On enrolment, the doctor and health educator see patients for thirty minutes each; patients on return visits spend approximately fifteen minutes each with clinical staff (either a doctor or nurse) and a health educator.

Routine laboratory tests are carried out at a local laboratory, which has been quality assured by MSF. At the first visit a urine dipstick (for white cells, red cells and protein) is performed. A routine set of blood tests is carried out: haemoglobin (Hb), kidney function tests (sodium, potassium, creatinine), fasting blood glucose, liver function tests (LFTs), cholesterol profile (total cholesterol and triglycerides), glycosylated haemoglobin (HbA1c) and microalbuminuria for known diabetics, and thyroid function tests (free thyroxine and thyroid stimulating hormone) for patients with known thyroid disease. Fasting plasma glucose and HbA1c are repeated at 3-monthly intervals for diabetic patients; urine dipstick is repeated biannually; and cholesterol profile, kidney function tests and microalbuminuria are repeated annually. The other named tests are repeated as required according to clinical protocols.

Medications are those that are considered standard of care in Jordan; most are on the MSF Green List, the list of medications approved for use in MSF projects, with some additions relevant to the local context. Medications are dispensed from the MSF pharmacy, located onsite at each clinic immediately following a medical consultation. Prescriptions are renewed at maximum one-monthly intervals. Patient data are maintained in paper-based purpose-designed chronic care files, which are stored securely at each clinic.

### 1.1.3 MSF NCD Clinical Guidelines

MSF-OCA has developed a new NCD Guideline to address the lack of standard guidelines on non-communicable disease management in humanitarian settings within MSF and elsewhere (http://hdl.handle.net/10144/618798). This provides programmatic and evidence-based clinical guidance for NCD management, addressing diagnosis; clinical examination; patient education regarding the disease, lifestyle modification and self-management of their illness; targets for disease control; medical management in including the assessment and management of global cardiovascular risk; management of acute and chronic complications; and onward referral. The conditions covered are: asthma, chronic obstructive pulmonary disease, diabetes, hypothyroidism, epilepsy, cardiovascular disease and hypertension, renal impairment, cancer and psychiatry. The guideline is currently being field-tested in MSF programmes in diverse settings but, to date, it has not been formally evaluated. Current indicators and outcome measures used by the programme are listed in Table 1 below.

**Table 1. Standard epidemiological and quality indicators for MSF NCD services**

### 1.1.4 Evolution of model of care

| **Epidemiological / service activity indicators** | **Quality indicators** |
| --- | --- |
| Number / % of new patient consultations per month | Number / % of DM patients that have micro-albuminuria or urinary protein testing in the last year (Target = 80%) |
| Number / % of follow-up consultations per month (% of total visits) (by age, gender, nationality) | Number / % of patients with DM or on ACE inhibitor (ACEi) with Creatinine testing in last year (Target 80%) |
| Number/ % of patients presenting with the following morbidities: DM Type I, DM Type II, Hypertension,  other CVD, Asthma, COPD, Hypothyroidism, other | Number/ % asthma and COPD patients who receive control review (spirometry or clinical) in last year (Target = 80%) |
| Number of new diagnoses over a period of time | Number/% of asthmatics with acute exacerbations/ admissions in last year (Target = 20%) |
| Number/ % of exits per month: % defaulter, % dead (by primary diagnosis),  % self-transferred, % other | Number / % of DM patients/ hypertensives with mean BP <= 140/90 over last 12 months  (Target = 80%) |
|  | Number / % of DM patients with mean HbA1C < 8.0 % (or average FPG<150) in last 12  months (Target = 80%) |

Programmatic changes over time include introduction of: (1) a home visit team (2) a humanitarian liaison officer and (3) a formal MHPSS service. Technical changes introduced over time include: (1) task shifting to nurses of follow-up consultations (2) introduction of MSF-OCA NCD Guidelines and (3) implementation of spirometry. Contextual changes that occurred since programme inception include: (1) introduction of co- payments by the Jordanian government for Syrian refugees accessing MoH primary care services in November 2014 (2) increased recruitment of vulnerable Jordanians to the MSF NCD programme in the last quarter of 2015 and (3) closure of the MSF NCD programme to new patients from May to October 2016.

### 1.1.5 Programmatic Changes over Time

Specific additions were made to the programme over time in response to patient and programmatic needs. A home visit service, which commenced in August 2015, comprises a doctor, nurse and driver. It operates six mornings a week and serves frail or immobile patients living within a ten-kilometre radius of the clinics. 150 patients have been enrolled. They are seen on a monthly basis initially; stable patients may be reviewed by phone and visited less frequently.

A humanitarian liaison officer (HLO) was employed in August 2015 to assist vulnerable patients with social issues and direct them appropriately to services provided by other humanitarian actors. Patients are referred to the HLO by the medical team or the mental health service.

A mental health component was added in April 2016 to serve patients with NCDs and relevant, identified mental health co-morbidities, which may impact on their NCD care e.g. bereavement, anxiety or depression. Doctors and nurses can refer patients to the service. Two trained counsellors provide mental health and psychosocial support (MHPSS) through group psycho-education and discussion sessions and individual counselling. Group sessions take place in clinic waiting rooms and content is responsive to issues raised by patients. Individual sessions support patients’ medical treatment. Formal feedback structures between counsellors and medical staff have been introduced and will undergo review and refinement.

### 1.1.6 Existing data sources, records and assessment processes

Routine cohort data are recorded in a paper-based patient file and entered daily into a purpose-designed MS Excel database by a trained data entry operator. The demographic data collected include: age, gender, mobile phone number, highest level of education; country of origin; refugee status and governorate of origin if Syrian; and insurance status if Jordanian. Data on Syrian refugees’ date of arrival in Jordan, UNHCR registration and Ministry of Interior card status are also collected if appropriate. Socio-demographic data are recorded on household size and crowdedness, type of dwelling and household makeup including gender and highest level of education attained by household head. Factors affecting treatment access and adherence are also documented i.e. impaired mobility and whether living with a treatment supporter.

Clinical data collected at the patient’s first visit include medical history, family history of relevant NCDs, lifestyle factors (smoking, alcohol consumption and exercise levels) and baseline vital signs (BMI, waist circumference, blood pressure, heart rate, blood sugar level and cardiovascular risk score assessment). New diagnoses; numbers, types, doses and reason to adapt medications; recommended specialist referral; and referral for laboratory tests are recorded at the first and each subsequent clinical visit.

At review visits, nurses record repeat vital signs and laboratory results; health educators record current exercise levels, smoking status and current stage of change on the Prochaska and DiClemente stages of change model for behaviour change. Symptom and functionality scores are recorded before and after a course of individual counselling, using Generalized Anxiety Disorder 7-item Scale (GAD7), Patient Health Questionnaire for Depression (PHQ 9) and a locally devised four-item screening tool for PTSD. These tools will be revised in early 2017.

Operational data recorded include numbers of new and follow-up medical consultations per month, type of consultation (nurse, doctor, home visit team), number of referrals made, number of health education sessions attended and programme exits (death, defaulter, voluntary defaulter). The number of MHPSS discussion groups, psycho-education sessions and individual counselling sessions undertaken per month are also recorded.

In addition, MSF-OCA has recently completed primary analysis of a cross sectional population based survey of Syrian refugees living in Irbid Governorate, undertaken for separate purposes. Data from this survey will provide initial information on the reach of MSF’s NCD activities and existing coverage of the target population.

Costing data are recorded in standard MSF pharmacy consumption tools, logistics supply tools, human resources and budgeting tools.

# 2. Study rationale, aim and objectives

There is a lack of data on models of NCD care and limited evidence on implementation, effectiveness and feasibility of NCD care in the humanitarian contexts where MSF works. The identification of NCD care as the greatest unmet health need amongst urban-based Syrian refugees living in Irbid, and rising NCD patient load in other settings, prompted MSF-OCA to develop a primary care based model of NCD care. This model is focussed on limiting treatment interruption and complications of established disease, and has evolved over time in response to clinical and programmatic needs. Lessons learned from Irbid may be adapted for MSF’s programmes in other contexts where NCDs are responsible for significant morbidity and mortality. The findings may also be used in MSF’s external advocacy work related to the scale-up of primary-level NCD care provision and cost of NCD medications in Jordan.

The overall aim of the study is to evaluate a primary care based model of NCD care delivered by MSF in Irbid, Jordan in order to refine the model and to generate evidence on its feasibility, acceptability and effectiveness with a view to translating a similar model to comparable humanitarian settings. This care model (outlined in section 1.1) will be evaluated through review of clinical indicators using routine cohort data; routine cost data; and qualitative data collected from patients, care providers and from key stakeholders.

The specific objectives are to examine the:

*Reach* (coverage) of the NCD service and its components to the intended target population.

*Effectiveness* of the NCD service. RE-AIM defines effectiveness as: the impact of an intervention on important outcomes, including potential negative effects, quality of life, and economic outcomes. Since this intervention has evolved over time, we cannot assess the effectiveness or cost-effectiveness of a specific intervention or strategy using a pre-post design and we do not have a comparator group. Thus, we defined “effectiveness” for this evaluation as quality of care. This will be examined by identifying trends in clinical outcomes and quality of care indicators; exploring perceived benefits, unintended consequences, behavioural outcomes and evaluating economic outcomes (See Table 2 below).

*Adoption / acceptance* of the NCD service and its components (including medication adherence) by the organisation, setting, staff and patients, and consequent changes to behaviour and practice.

*Implementation* of the NCD service and its components including fidelity in terms of consistency of applying the defined guidelines and processes; guideline usability, adaptation of structures, processes and tools; and costs.

*Maintenance* of the NCD service and its components in patients, programme and organisation over time.

# 3. Methods

## 3.1 Description of the study sites

The study setting will be Irbid city in northern Jordan. Irbid governorate, with a population of over 1.7 million, borders southern Syria. 27% of the 1.27 million Syrians living in Jordan are located in Irbid governorate ^18^; of these, 135,280 individual Syrians are registered with the UNHCR as persons of concern ^4^.

The majority of registered Syrian refugees in Jordan live outside official refugee camp settings within the local community ^4^. Until policy changed in November 2014, non-camp-dwelling UNHCR-registered refugees were entitled to free primary healthcare, which was funded by UNHCR and delivered through the Jordanian public health system ^19–21^ . The Jordanian system consists of a mix of public and private healthcare. Public care is free to three categories of insured citizens: civil servants, military and those categorised as “destitute” ^22^. While camp-dwelling refugees are still entitled to free primary healthcare, out-of-camp registered refugees now pay a subsidised fee, approximately 35-60% of the cost, similar to that paid by uninsured Jordanians ^21,23^.

MSF and other non-governmental organisations support the Jordanian government and the UNHCR in providing health care for non-camp-dwelling refugees. However, evidence from repeated surveys suggests that, as demand exceeds current capacity, both access and costs have proven barriers to refugees accessing essential health care and medications ^20,21,24^. Currently, MSF is the only actor providing free, specialised NCD care in Irbid governorate.

The previously completed MSF household survey was conducted in Irbid governorate in northern Jordan. Most of the area is uninhabited, mountainous terrain. The majority of Syrian refugees live in urban and peri-urban areas of Irbid city or Ramtha town, while the remaining 40% live in rural areas.

This evaluation, including use of routine service data, will take place in the two vertical, primary care based MSF-supported clinics providing NCD care in Irbid city centre. The first clinic was opened in December 2014 within an MoH primary care facility in Ibn-Sena. The clinic is located on the first floor, accessed by stairs only. The second clinic opened in April 2015, alongside a private organization, Arab Medical Relief (AMR), in their facility at Ibn-Rushd. This is located in the basement of a major civic building, also accessed by stairs only. Both clinics may be reached by public transport. Each contains a reception desk, central waiting area, triage room, individual consultation rooms, a larger room for group education sessions and a pharmacy. Ibn-Sena also has a treatment room where diabetic foot care is carried out.

**Table 2. List of main indicators and data method/source based on RE-AIM**

| Objective (Questions) | Domain | Indicator | Methods  (a single methodology may feature under several RE-AIM headings) |
| --- | --- | --- | --- |
| Reach  Does the programme reach its target population? | Coverage | Target population prevalence of diabetes & CVD  Number of people eligible for care for diabetes & CVD (inclusion/exclusion criteria)  Number of people receiving care for diabetes & CVD  Representativeness of those reached  Prevalence of NCD and identified, relevant MH comorbidity; eligibility for MHPSS services; numbers referred/receiving care; representativeness of those receiving MHPSS service | Existing cross-sectional survey previously conducted by MSF  Routine facility cohort data  2 focus groups with patients  Approximately 16 semi-structured patient interviews  Key informant interviews |
| Adoption/ initial acceptance  Is the MSF model of NCD care accessible and acceptable to patients, providers, organisation and community?  Is the MSF NCD guideline acceptable to staff? | Participation | Description of intervention location, cadres of staff and qualifications; inclusion/exclusion criteria of staff/settings delivering service  Sources and perceptions of information and support for participation in NCD service & components (e.g. HE, MHPSS)  Experience of receiving and providing NCD care including use of clinical guideline  How programme participation has influenced patient/staff well-being and staff ways of working. | Routine facility cohort data  2 Focus groups with patients  Semi-structured interviews with approximately 12 clinic staff and 16 patients |
|  | Accessibility/ acceptability | Duration and frequency of NCD service and components  Patient characteristics of those accessing individual programme components (e.g. HE, HLO, MHPSS, foot care)  Staff (e.g. ratio of staff per patient)  Structures and tools  Treatment continuity/rupture  Staff and patient perceptions of availability and accessibility / barriers to access of service components (clinical review, HE, HLO, MHPSS, HV, Foot care)  Staff perspectives on acceptability / usability of NCD guideline  Key stakeholder views on acceptability and accessibility of MSF NCD service and components (e.g. MSF, MoH, NGO staff,)  Self-reported medication adherence levels and medication beliefs | Routine facility cohort data  Key informant interviews  Participant observation  2 focus groups with patients  Semi-structured interviews with approximately 12 clinic staff and 16 patients  Self-report medication adherence questionnaire |
| Implementation  To what extent was the intervention delivered as intended?  What are the facilitators and barriers to implementing the programme from a patient, provider and programmatic perspective?  What are the essential components and adaptations necessary to delivering an NCD service in this setting?  What are the start-up and incremental costs of delivering such a service? | Fidelity of programme delivery  (Process Indicators;  indicators in bold also reflect quality of medical care) | Extent to which clinical guideline delivered as intended:  Number / % of eligible patients with HTN with annual FPG performed during the reporting period  Number/ % of eligible patients with diabetes that have had an annual foot check/ eye check performed during the reporting period  Number / % of DM patients that have micro-albuminuria or urinary protein testing during the reporting period  Number / % of DM patients on ACE inhibitor (ACEi) with Creatinine testing during the reporting period  Number/ % asthmatics and COPD with control review (spirometry or clinical) during the reporting period  Number / % of active cohort attending a health education session at last clinical visit within reporting period  Number of MHPSS group sessions taking place monthly during reporting period  Number/ % of referred patients attending MHPSS individual counselling sessions  Number/ % of times when clinical action taken based on clinical or laboratory findings according to guideline (see Annexe 2d for detail) | Clinical audit  Participant observation  Routine health facility cohort data |
|  | Adaptations | NCD care adaptations to the local setting (e.g. cultural adaptations; dietary and exercise, smoking advice)  Programme adaptations related to humanitarian setting and role e.g. response to patients’ psychosocial needs and NCD-relevant mental health co-morbidities | Key informant interviews  Participant observation  Semi-structured interviews with 12 MSF/clinic staff and 16 patients |
|  | Cost | Staff time  Start-up and recurrent implementation costs (indirect, intermediate and final cost centres)  Average unit costs and unit costs stratified by morbidity) | Key informant interviews  2 Focus groups with  Medicine/supply/ staff costs  Staff time estimates |
| “Effectiveness”/ Quality of Care  What are the trends in clinical outcomes and quality indicators of the programme?  What are the perceived benefits/unintended consequences from a patient and provider perspective? | Clinical Outcomes | No./% patients with hypertension that have a most recent BP <= 140/90 6 and 12 months post enrolment and trend from baseline  Number / % of patients with diabetes that have a most recent BP <= 140/90 6 and 12 months post enrolment and trend from baseline  Number / % of patients with diabetes with last HbA1c < 8.0 %/ 7.0 % 6 and 12 months post enrolment and trend from baseline  Number/% with a reduction of >= 0.5 mmol/L in cholesterol level from baseline 6 and 12 months from enrolment  Number/% of patients with asthma / COPD free from exacerbations/ admissions in the previous 6 months  Number / % of patients who report decreased/quitting smoking within reporting period  Number / % of patients who report increased levels of exercise from baseline during reporting period  Trend recommended referrals to another facility for acute complications/specialist care, as a proportion of active cohort | Routine facility cohort data  Costing data (for incremental costing analysis)  2 Focus group with patients  Semi-structured interviews with approximately 12 clinic staff and 16 patients |
|  | Quality Indicators | Proportion of recommended referrals to other services that are appropriate as per guideline  Number/ % of active patients with CVD prescribed a statin during reporting period  Number/ % of patients with CVD prescribed aspirin during reporting period  Number/ % of patients with CVD prescribed at least one anti-hypertensive during reporting period  Number/% of patients with COPD/asthma with inhaler technique check documented  Number/ % of times when appropriate clinical action taken based on clinical or laboratory findings according to guideline (see Annexe 2d for detail)  Trend in defaulters as a proportion of active cohort during reporting period  Description of cohort deaths (patient characteristics) | Clinical audit  Participant observation  Routine health facility cohort data |
|  | Perceived Effectiveness | Patient and providers perspectives on effectiveness of programme components (clinical review, medications, HE, HLO, MHPSS, HV) | Key informant interviews  2 Focus groups with patients  Semi-structured interviews with 12 MSF/clinic staff and 16 patients |
| Maintenance  What are the challenges and facilitators for patients to remain in the programme?  What are the costs involved in maintaining the programme?  What are the programmatic challenges and adaptations made to maintain the programme? | Individual Level | Number/% of patients active 6 months post enrolment in reporting period  Numbers of medications and daily pill count at last consultation during reported period  Self-reported medication adherence rates and medication beliefs  Qualitative measure of individual-level maintenance:  Key challenges in maintaining medical treatment (including medication concordance)  Key challenges in altering lifestyle (diet, exercise, smoking)  Key mental health/ psychosocial challenges  Types of support available and strengths and challenges of the support (health education, MHPSS, HLO, family and community support) | Routine facility cohort data  Clinical Audit  Key informant interviews  2 Focus groups with patients  Semi-structured interviews with 12 MSF/clinic staff and 16 patients  Medicine/supply/staff costs  Staff time estimates  Self-report medication adherence questionnaire |
|  | Organisational Level | Measures of cost of maintenance  Institutionalisation of the programme/modifications made for maintenance  Alignment with organizational mission |  |

## 3.2 Overall study design

A mixed-methods design will be used, based upon the Reach Effectiveness Adoption Implementation Maintenance (RE-AIM) framework (see <http://www.re-aim.hnfe.vt.edu/>). RE-AIM is a widely used framework to evaluate the impact of public health interventions by assessing five key domains: reach, effectiveness, adoption, implementation, and maintenance. The research methods will include a routine cohort data design, secondary analysis of pre-existing cross-sectional survey data, clinical audit, self-administered medication adherence survey, qualitative research and use of routine service data. This mixed methods approach is being used in order to address the study objectives within the overarching RE-AIM framework. Data from the quantitative and qualitative portions of this study will be triangulated and presented in a descriptive report. Further details on the study indicators and data sources, analysis and management are described in detail under each methodology section below and are summarised in Table 2 and Figure 1.

## 3.3 Study population

The study population for this evaluation consists of four groups:

1. The broader study population, which was included in the pre-existing, completed cross-sectional household survey, consisted of UNHCR-registered and unregistered Syrian refugees living outside of official camp settings within Irbid governorate.

2. Patients enrolled in MSF’s NCD clinics:

This refers to non-camp-dwelling Syrian refugees and vulnerable Jordanians who have been enrolled in MSF’s vertical NCD clinics in Irbid, Jordan, since patient registration was introduced in December 2014. To date (November 2016), 4672 NCD-related patients (aged 1 to 94 years) have been registered in the two clinics. Inclusion criteria are (1) Socio-demographic [Syrian refugee, refugee from another origin or vulnerable Jordanian (defined as patients who were identified as vulnerable according to the Ministry of Social Development National Aid Fund list)] and (2) Medical [confirmed CVD (including angina pectoris, myocardial infarction, ischaemic stroke, peripheral vascular disease), CVD risk factors (HTN), COPD, Asthma and/or DM]. Patients must fulfil both criteria.

The routine cohort data analysis, including the clinical audit, will include data from all enrolled patients aged six years and above (using files of patients admitted from January 2015 through to December 2016); qualitative data gathered through focus group discussions (FGDs) and semi-structured interviews (SSIs), and data from the adherence survey will be collected from a sample of adult patients aged 18 or older.

3. Clinic staff:

Semi-structured interviews will also take place with current and former healthcare workers and MSF management staff who provide(d), supervised or supported MSF NCD services in Irbid, Jordan.

4. Key Stakeholders:

Informal interviews will take place with key stakeholders, including members of the Jordanian Ministry of Health; Irbid District Health Office; clinical manager of the Ibn Sena MoH clinic where MSF‘s clinic is co-located; and Syrian refugee community leaders.

The eligibility criteria, recruitment and sampling methods are described further under each methodology’s sub-section below.

## 3.4 Cohort study method

The cohort design seeks to describe the patient cohort and explore clinical and quality of care outcomes of the NCD programme by analysing routine clinical data and performing a clinical audit of patient files from the two clinics. This evaluation does not seek to formally evaluate programme effectiveness since this programme has evolved over time and there is no single intervention that can be evaluated nor do we have a comparator group. Thus, we have chosen to evaluate to what extent the programme is: a) consistently implementing the evidence-based clinical guideline which incorporates interventions known to reduce mortality and morbidity e.g. appropriate statin prescribing, and b) achieving evidence-based targets for disease control e.g. blood pressure control. The cohort study will also include descriptive costing analysis.

### 3.4.1 Cohort study area and study population

The study location will be the two MSF-OCA supported clinics in Irbid city, northern Jordan. The first clinic was opened in December 2014 within an MoH primary care facility in Ibn-Sena and the second, opened in April 2015, works alongside a private organization, Arab Medical Relief (AMR), in their facility at Ibn-Rushd.

To date (November 2016), 4672 NCD-related patients (aged 1 to 94 years) have been registered in the two clinics, since patient registration was introduced in December 2014. Inclusion criteria are (1) Socio-demographic [Syrian refugee, refugee from another origin or vulnerable Jordanian (defined as patients who were identified as vulnerable according to the Ministry of Social Development National Aid Fund list) and (2) Medical [confirmed CVD (including angina pectoris, myocardial infarction, ischaemic stroke, peripheral vascular disease), CVD risk factors (HTN), COPD, Asthma and/or DM Type I or II]. Patients must fulfil both criteria. All NCD patients aged six years and older with at least two recorded visits (to enable evaluation of outcomes) will be included in the study cohort. This includes any new patients presenting with a new or existing diagnosis of a target NCD.

### 3.4.2 Cohort study main outcome measures

The principal **clinical outcome measures** for patients in the cohort are:

DM: HbA1c < 7 or 8%; proportion of patients meeting individualised target at last visit within the reporting period and trend from baseline

Hypertension: BP < 140/90 mmHg: proportion of patients at target at last visit within the reporting period and trend from baseline

Cardiovascular disease: change in total cholesterol level from baseline

Asthma / COPD: Free from exacerbations / admissions in last six months

The principal **quality of care outcome measures** are the following:

Number/ % of active patients with CVD prescribed a statin during reporting period

Number/ % of patients with CVD prescribed aspirin during reporting period

Number/ % of patients with CVD prescribed at least one anti-hypertensive during reporting period

Number/% of patients with COPD/asthma with inhaler technique check documented

Number/ % of times when appropriate clinical action taken based on clinical or laboratory findings according to guideline (see Annexe 2e for detail)

Description of trends and patient characteristics in programme defaulters and deaths

Proportion of recommended referrals to other services that are appropriate as per guideline

Death is defined as confirmed death reported by family or community member. A defaulter is defined as a patient who has not attended any NCD clinic appointments within the last 90 days, is not known to have died or moved out of the area, and has not been successfully contacted by phone on two occasions by the clinic registrar. The definition was changed in October 2016 to reflect the newly extended appointment interval for stable patients to: two consecutive missed appointments; not known to have died, moved out of the area, or become immobile and ineligible for home care; and has not been successfully contacted by phone on two occasions by the clinic registrar.

Exacerbation of respiratory condition refers to self-reported or documented temporary, usually reversible, worsening of the condition requiring a step-up in treatment or hospitalisation, depending on severity. Acute referral to specialist care or to the emergency room is recommended by the NCD guideline when the condition is not controlled by the maximum available primary-level treatment; additional secondary-care level diagnostics or treatments are required; in an uncontrolled acute exacerbation; or in cases of clinical uncertainty. Specific referral criteria for each condition are further described in the guideline.

Other outcome measures are listed in Table 2 (page 26).

### 3.4.3 Cohort data sources

The cohort will use retrospective routine facility data collected from files of patients admitted from the start of January 2015 to the end of December 2016. Data on costs for the economic analysis will be obtained from logistics supply, accounting and pharmacy records, and from time estimates obtained through observation and discussions with staff (see below for more details).

### 3.4.4 Cohort data management

Paper data are collected on a daily basis by existing MSF data clerks and any abnormal or missing values are discussed with the nurse or doctor and both paper and electronic data updated accordingly. Single data entry is performed by a trained data entry operator using a password-protected Excel software database developed for the programme. The local MSF epidemiologist will analyse the routine service data for the cohort study and will ensure the quality of data collection and entry through weekly checks. The data is also emailed to the MSF-OCA NCD advisor on a monthly basis for further quality checks.

The routine cohort data are held on password protected databases and PCs in a secure location. Data used for the cohort analysis will be extracted from the database and anonymised, with removal of unique identifiers other than the MSF identification number. Data on costs for the economic analysis will be held on a secure Excel file.

### 3.4.5 Cohort data analysis

Cohort data from both clinics will be aggregated and will be analysed using RStudio v1.0.136 (RStudio, Boston, MA 02210, USA). Descriptive statistics will be used to describe the patient cohort, explore clinical outcomes and analyse trends in outcome measures from baseline. To attempt to account for programmatic changes that occurred since the intervention began, comparison will be made between patients who entered the programme during different periods. Analysis will include proportions and means, presented with 95% confidence intervals (95%CI). Differences in proportions will be measured using Pearson χ2 test while differences in means will be measured using two-sample t-tests, presented with p-values. Clinical outcomes will be compared using multiple linear regression to explore the effect on key clinical outcomes of age, gender, origin (Syrian or Jordanian), economic status, mobility, type and number of morbidities (including relevant, identified mental health co-morbidity), and number of medications/pill burden.

### 3.4.6 Clinical audit

A paper-based checklist (Annexe 2d) will be used by a trained clinical staff member(s) to evaluate a sample of two hundred patient files with ten per cent of the sample cross-checked by the medical supervisor. A randomly selected sample of files of patients enrolled in the programme for over 12 months will be used. In addition, 65 files (based on a precision of 10%) from amongst the patients with asthma will be randomly selected, since the active cohort contains fewer than 300 patients diagnosed with asthma. The data will be anonymised, entered by a trained data entry operator into a password-protected Excel software database, analysed using proportions measured with Pearson’s χ2 test and reported with 95% confidence intervals.

### 3.4.7 Costing analysis

A descriptive costing analysis will be undertaken to investigate total and unit costs, changes in these costs over time and the incremental costs of adding different components to this model of NCD care. We will describe the costs of implementing routine clinical review, providing medications and specific diagnostics e.g. spirometry, and the incremental costs of implementing the health education; mental health and home visit elements of the programme. Using an ingredients-based approach, the start-up, recurrent and capital costs will be determined, including the cost of supplies, room rental, transport, staff time and staff training. The cost analysis will take a health services perspective and will consider direct costs of alternative models of care.

Cost data for the drugs and supplies will be collected from routine accounting records and supply orders recorded in standard MSF budgeting and supply tools held by the MSF office in Irbid. Estimates of staff time spent on care and training will be collected by research staff through observation of care services in the two MSF clinics and discussions with relevant staff using a standardised methodology for calculating time estimates. These time estimates will then be converted to monetary values based on salary data held by the MSF office in Irbid.

## 3.5 Self-administered medication adherence questionnaire

The objective of this small exploratory study is to obtain a rapid assessment of patients’ medication adherence and beliefs. A secondary objective of this small-scale study is to explore the feasibility of employing this questionnaire in terms of time, usefulness and performance. This rapid assessment tool should provide insight into medication adherence while imposing minimal burden on respondent and data collector and thus may prove useful for repeated administration within the Irbid programme and more broadly within MSF.

### 3.5.1 Adherence questionnaire methodology

A self-administered questionnaire using translated existing tools will explore patients’ behaviours and beliefs regarding medication adherence.

### 3.5.2 Adherence questionnaire population

The study location will be the two MSF-OCA supported clinics in Irbid city, northern Jordan. Any patient enrolled in either clinic according the criteria described in section 3.4.1, aged eighteen years or older, has attended the clinic for over one month and has had at least one admission and one review medical consultation, is eligible to take part in the adherence survey.

### 3.5.3 Adherence questionnaire eligibility criteria, recruitment and sampling

A randomly selected sample (e.g. through randomisation of patient numbers) of MSF patients aged eighteen years or older attending either MSF clinic over a two–week data collection period, will be approached to fill the 17-item questionnaire, in order to give a sample size of 300 (based on previous studies ^25^). Questionnaires are presented in Arabic and should take approximately fifteen minutes to complete. The Arabic-fluent data collector will administer the questionnaire to patients with limited literacy and this will be recorded on the questionnaire.

### 3.5.4 Adherence questionnaire data tools

The questionnaire combines the Medication Adherence Report Scale (MARS-5) and the Beliefs about Medicines Questionnaire-Specific (BMQ-S) (See Annex 2f). These have been selected as they are derived from and validated in chronic disease populations and also incur no license fee ^25^.

The MARS-5 (Horne et al., 2002) is a 5-item, self-report instrument assessing a range of non-adherence measures. Respondents are asked to rate the frequency they engage in each of the adherence-related behaviours on a five-point scale, where 5 = ‘never’, 4 = ‘rarely’, 3 = ‘sometimes’, 2 = ‘often’ and 1 = ‘always’. Scores for each item are summed to give a total score; higher scores indicate higher levels of reported adherence. The MARS-5 may be used to distinguish between intentional and unintentional adherence, which may guide intervention strategies to improve adherence (Horne, R. personal correspondence). Permission to use the MARS-5 was granted by the authors of the original English ^26^ and validated Arabic versions ^27^.

The BMQ-S consists of 10 statements about medications scored using a 5-point Likert scale (from 1 = strongly disagree to 5 = strongly agree). Five questions relate to the perceived necessity of medications and five relate to patients’ concerns about taking medication ^28^. Permissions to use the BMQ-S were granted by the authors of the original English ^28^ and validated Arabic versions ^27^.

### 3.5.5 Adherence questionnaire data management

Data will be anonymised and entered into a purpose-designed, password-protected Excel database by a trained data entry operator.

### 3.5.6 Adherence questionnaire data analysis

The originators of these scales require that they are analysed and reported in accordance with their instructions in order to promote uniformity and facilitate comparison of findings across studies. Results will be analysed using basic descriptive statistics. Analysis will include proportions and means, presented with 95% confidence intervals (95%CI). Adherence rates will be compared using multiple linear regression to explore the effect of age, gender, civil status, education status, type and number of morbidities, number of medications and whether administered by self or by the data collector.

## 3.6 Cross-sectional household survey data analysis

This survey, undertaken in June and July 2016, was led by the MSF-OCA Irbid NCD Project team and received MSF-OCA ERB approval. The primary objective was to determine the level of access to health care services for Syrian refugees living out-of-camp in Irbid governorate, Jordan. Secondary objectives included: a) describing the socio-demographic characteristics, economic circumstances and health care utilisation of the surveyed population, b) estimating coverage of essential health services, particularly MSF-provided services and c) estimating health service needs and barriers to health care access amongst the surveyed population.

### 3.6.1 Household survey population

The study population consisted of UNHCR-registered and unregistered Syrian refugees living outside of official camp settings in Irbid governorate.

### 3.6.2 Household survey eligibility criteria and sampling

A person was included in survey if he or she met the following criteria: living in a selected household; arrived in Jordan in or after January 2012 and at least 6 months prior to the survey interview; and permission had been granted to take part in the interview by the household head. To be eligible to answer specific health questions the following criteria were also required: general health: adult aged 18 years or older; NCD care: adult 18 years or older and living with one or more of the following conditions: hypertension, CVD, diabetes type I or II, chronic respiratory disease, thyroid disease or cancer; child health: under five years of age; antenatal care: female aged fifteen to forty nine years and had given birth to a live infant in the previous twelve months in Jordan.

Sample size was calculated using OpenEpi and STATA 13 (StataCorp, College Station, TX, USA), using reference values based on UNHCR household surveys^29^. An estimated number of 2616 households needed to be interviewed, sampled in 327 clusters of eight households. A two-stage cluster sampling methodology was adapted from the standardised methodology recommended by the WHO (Henderson and Sundaresan 1982).

### 3.6.3 Household survey data collection and management

Data were collected by trained interviewers using an electronic tablet-based survey tool. Data were uploaded daily to an off-site server using secured MSF Wi-Fi. Aggregate data were exported daily for back-up and were stored on a password-protected computer.

### 3.6.4 Household survey data analysis

Primary data analysis has been completed using descriptive statistics and multivariate regression modelling and will be reported elsewhere. Secondary analysis of the pre-existing data using RStudio v1.0.136 (RStudio, Boston, MA 02210, USA) will determine the number of people eligible for and receiving NCD care, the geographical reach of the programme and the target population’s knowledge of MSF. Results will be analysed using basic descriptive statistics, including Pearson’s χ2 test for proportions and will be presented with p-values.

## 3.7 Qualitative research

The objective of the qualitative research is to explore key issues related to adoption, implementation and maintenance of the intervention, with a particular focus on the both the NCD guideline implementation and the MHPSS service.

### 3.7.1 Qualitative methods

Focus group discussions, semi-structured interviews, key stakeholder interviews, and participant observation will be used. Two same-sex focus groups will be conducted with NCD patients. These will explore the reach and implementation of the NCD model (e.g. perceived benefits, acceptability, barriers, unintended consequences). Topics will include examination of specific components of the NCD care (e.g. service provision, clinical consultation, medication adherence, health education, MHPSS and/or support from the HLO, the home visit service). These focus groups will then be followed at a later date (e.g. the following week) by semi-structured interviews with purposively selected focus group participants and other patients in order to explore further key issues raised in the focus groups, while ensuring that patients accessing each element of the service are represented.

Semi-structured interviews will also be conducted with health care staff involved in the NCD services. These will focus particularly on issues related to: (i) reach (e.g. available resources and support, perceived benefits versus costs, barriers, suggestions for improvement; and (ii) adoption and implementation (e.g. values and capacity, implementation challenges and adaptations, compatibility with values, resources, complexity); (iii) maintenance: support, activities to support sustainability, perceived benefits vs. costs. The interviews will include a focus on specific components of NCD care (e.g. service provision, clinical consultation, medication prescription and adherence, health education, MHPSS and/or support from the HLO, home visit service). Key stakeholder interviews will be conducted with Syrian refugee community leaders, representatives from the MoH and other NGOs. Participant observation will be undertaken at clinics.

### 3.7.2 Qualitative research study population and sampling

Information about the study will be provided to all patients attending both MSF NCD clinics in Irbid using posters and leaflets and discussion during group education and counselling sessions. Two same-sex focus groups of approximately eight people each will be conducted with a cross-section of NCD outpatients, aged 18 or above, including a mix of patients attending either of the two MSF clinics. Patient selection will be based on NCD type and severity, age, gender and literacy as suggested by MSF clinical staff.

These focus groups will be followed by individual semi-structured interviews. Some participants of the focus groups who had particularly insightful perspectives will be purposively selected for individual interviews by the principal investigator and local study coordinator in order to follow-up on key issues raised. Specific permission to re-contact focus group participants and the right to withdraw this consent will be included in consent forms. The research coordinator will separately record the participant’s preferred means of contact to protect their confidentiality.

Patients attending either clinic will be approached in the waiting room by the local research coordinator, informed about the study and invited to participate in focus groups and /or individual interviews. Approximately 12 or more individual interviews with NCD patients recruited from both clinic sites will be conducted, aiming to include patients who have accessed each component of the NCD service (including HLO, home visit service, MHPSS). This is subject to principles of saturation.

Approximately 12 semi-structured interviews will be conducted with key staff involved in the provision of NCD care in either of the two MSF clinics. These staff will be purposively selected to reflect the range of activities and staff functions involved. They may include the doctors and nurses, psychosocial workers, field co-ordinator, and expatriate MSF medical and research staff in Irbid and internationally either currently or previously involved in the NCD programme. Participants will be eligible if they are full-time members of staff (i.e. not temporary workers) and have worked in the NCD clinic, in the MSF Irbid NCD project office or as MSF Jordan country coordination or headquarters staff from December 2014 to the present for a minimum of three months.

Approximately six key stakeholder interviews will be conducted with Syrian refugee community leaders and with representatives of the Ministry of Health and District Health Office who are involved in NCD care delivery. The community leaders, identified during the MSF household survey and through the MSF Irbid team’s community engagement work, will be invited to an interactive information meeting where they will be invited to participate in interviews. If more than three people volunteer, three names will be selected at random using Microsoft Excel.

### 3.7.3 Qualitative data collection

The focus groups will last approximately two hours and the individual semi-structured interviews will last approximately one hour. Separate topic guides will be used for the focus groups and interviews with patients, service providers and key stakeholders (see Annexes 2 a-d for draft topic guides). These topic guides have been adapted to the context by the principal investigator who has experience of working in NCD clinics with Syrian refugees in Jordan and in other contexts. The included topic guides are indicative only; they will be further refined based on findings from the focus groups and from two pre-test interviews, which will be conducted to improve internal validity. The finalised topic guide will seek to provide guidance and structure to the interviews but not to restrict the discussion. They will be translated into Arabic and back translated into English.

The focus group and individual interviews with NCD patients, and interviews with key health staff are expected to take approximately 4 weeks, and will take place in August 2017. Training will be provided to the local researchers who will facilitate the focus groups and conduct interviews with NCD patients and some health care staff.

The focus group and interviews will be conducted either at the MSF clinics or office, or by telephone or Skype for expatriate workers who have left Irbid. The focus groups and interviews with NCD patients will be conducted in Arabic by either a male or female fluent Arabic speaker as appropriate (the local MSF research coordinator and one additional trained local interviewer). The non-Arabic speaking principal investigator will be present as moderator during the focus group discussions and will require a translator. Both focus group discussions and individual interviews will be audio recorded, typed as transcriptions in Arabic and then translated into English. The interviews with health staff will be conducted in Arabic by the local MSF research coordinator or English by the principal investigator, as appropriate, and audio recorded where possible (unless conducted by telephone or Skype where notes will be used instead).

Key stakeholder interviews will be informal and will last approximately one hour. They will be conducted in Arabic by the local research coordinator or in English by the principal investigator, as appropriate, and will take place at a location convenient to the interviewee e.g. MoH office either in person or via Skype and will be audio-recorded.

Participant observation will be conducted by the principal investigator with a translator. It will include observation of working practices, health education and group counselling sessions (including use of MSF OCA clinical guidelines, data recording interaction between staff and patients, and patient participation). Clinic layout, organisation, staffing level, staff time expenditure and patient flow will also be observed. Field notes will be taken and transcribed into a password-protected computer record. These will be triangulated with other data sources including data from FGD and SSIs and costing data.

The qualitative research will be led by an expatriate qualitative researcher (the principal investigator) who will help finalise the study design and instruments, be involved in the focus groups and interviews, undertake the participant observation and lead the analysis and write-up of outputs. This will be conducted in collaboration with a local research coordinator from within the MSF field team. Bayard Roberts and Pablo Perel (LSHTM) will provide advisory support.

### 3.7.4 Quality assurance

The quality of the interviews will be assured in the following ways: First, the interviews will be led by researchers (i.e. PI and the local MSF research coordinator) who are experienced in qualitative research methods. Second, the interview topic guides will be developed following a rigorous process of expert consultation, group discussions and pre-testing to ensure relevance, reliability, and appropriateness. Third, the quality of the data transcription and translation will be assured through random checks and discussions with key staff involved in the data collection. Experienced transcribers and translators will also be used, and training given to them on the research to ensure familiarity with key terms and concepts. Fourth, the data analysis methods and use of specialist coding software (NVivo) will support transparency and reliability in the analytical decision-making (see below). This will be supported through triangulation (e.g. with other data sources) and peer review and discussion. Fifth, a limitations section will be included in any project outputs to clarify limitations in the study. Sixth, regular meetings will be held between the researchers to discuss and maintain quality assurance. Finally, the qualitative research will be underpinned by agreed principles of good practice including transparency, comprehensiveness, reflexivity, ethical practice, being systematic.

### 3.7.5 Qualitative data analysis

A priori themes based on the NCD literature will be identified and included in topic guides (Annexes 2 a-d). A computer-assisted qualitative data analysis software package, NVivo 11 (QSR International), will be used to manage the qualitative data. Thematic analysis, using a positivist approach, will be utilised to detect key emerging themes arising from the data, while using the overall RE-AIM framework to help structure it. Where appropriate, the qualitative data will also be compared and contrasted with the other data sources, including quantitative data from the cohort study and costing data, to triangulate study findings.

Figure 1 Irbid NCD Evaluation Summary of Methodologies and Indicators (reproduced and adapted from De Silva *et al* ^30^)

# 4. Ethical considerations

## 4.1 Social value

Potential patient benefits from the cohort include improved continuity and quality of NCD care and reduced risk of acute and chronic complications. Community-level benefits include access to improved NCD care in Irbid governorate. Potential programme benefits include provision of new information to help understand the strengths and weaknesses of the NCD service and its potential for use in other settings. The study team will ensure that any changes to NCD programming and operations suggested by this research are implemented within MSF projects in Jordan, in the wider Syrian refugee context and elsewhere; and that the findings inform planning for subsequent NCD interventions within MSF programmes.

There is currently a lack of evidence regarding the delivery of NCD interventions in crisis settings, particularly on longer-term outcomes. National and international level benefits of this evaluation include adding to the broader knowledge base by identifying implementation bottlenecks; exploring programme feasibility, cost and use of a setting-specific guideline; and identifying future research questions. Findings from this study will be available to the Jordanian MoH, NGOs and other relevant actors who are involved in the planning and implementation of similar programmes.

## 4.2 Potential risks from the study

The focus groups could discuss emotional challenges of having NCDs, which could possibly cause distress. Overall, these risk are expected to be low and the programme has experienced psychosocial workers to respond to any participant distress. The local qualitative research coordinator will receive training in interview technique, specific briefing on refugee mental health and debriefing post-interviews. Our previous experience of conducting interviews on this topic at the MSF hospital in Mweso in DRC did not report any cases of distressed respondents.

## 4.3 Respect for and protection of recruited participants and study communities

Acknowledging the time burden and potential direct and indirect costs that focus group discussion and interview participants may incur, the study will provide transport costs and refreshments during the process. Interview participants will be able to select a time convenient to them to minimise potential loss of earnings.

Prior to the study, the research team will meet with Ministry of Health co-investigators and other relevant staff to discuss the study with them (including its aims, objectives, methods and process) and gain their support for the study. The Jordanian and broader research community will be engaged through presentations at conferences, scientific meetings and through scientific publication.  In addition, local medical staff members will gain research skills through their contribution to this evaluation.

The Irbid NCD programme initiated a community engagement strategy in early 2017.  Syrian refugee community leaders were identified during the MSF Syrian refugee household survey conducted in 2016 and through the new outreach activities. These community leaders will be invited to an interactive meeting in advance of the focus groups discussions and individual interviews. Their opinions and concerns will be elicited and will inform the topic guides used. The community leaders will also be invited to participate in key stakeholder interviews.

## 4.4 Informed consent

Since the cohort study is based on analysis of routine patient data it will not involve any additional procedures and patients in the cohort study will not be asked to give written consent for use of their routine data. However, information sheets on the walls of the clinic will inform patients about the study.

For the completed MSF cross-sectional household survey, written informed consent was obtained prior to the beginning of each interview. For illiterate individuals or in case the interviewee agreed to conduct the interview but did not want to sign, verbal consent was obtained and testified by the data collectors. Further, for every subsection, which interviewed different members of the household, verbal consent was obtained and recorded individually for every section.

Participants in the qualitative study and medication adherence survey will be asked to give written consent to participate. If the individual’s literacy is limited, they can give verbal consent provided that a witness who is appointed by that individual can sign the consent form on their behalf. For the participant observation component of the study, participants will receive the information sheet; they will be asked to give verbal consent and they will be advised they can opt out of participating at any time without repercussion.

For securing written consent from former health care workers who are no longer working in Irbid and who will be interviewed telephonically or via Skype, the information sheet and consent form will be emailed to them in advance of the interview, and written consent sought through email confirmation. Verbal consent will also be sought prior to the telephone/Skype interview.

The contents of the information sheet and consent form are given in Annex 1. These contain key elements related to: the purpose of the study and qualitative methods; the process and summary content of the interviews; the respondent selection process, the potential harms from the study; and clarification that the potential benefits from the study will be for knowledge and clinical practice generally, and not for the participants own health status (in order to avoid therapeutic misconception); the risk of residual disclosure, the ability to pause or stop at any point, confidentiality, anonymity, and freedom of consent.

## 4.5 Safety considerations

To address any inappropriate or harmful prescribing potentially identified during the evaluation, the patient will be informed, the error corrected and any identified harm treated as appropriate. In addition, a comprehensive audit will be undertaken to identify and remedy similar errors and refresher training sessions undertaken with staff.

To avoid therapeutic misconception, the written information sheets and verbal information will make it extremely clear that participation in the research will have no influence on the treatment received or clinical outcomes (see Information Sheet in Annex 1).

To avoid the risk of residual disclosure in focus group discussions, interviewers will not be aware of participants’ specific disease status and so will not be able to accidentally disclose it or give out information for other participants to be able to deduce the stats of fellow participants. Participants will also be reminded about the risk of residual disclosure in the information sheet and verbal introduction prior to the focus group.

To avoid NCD clinic healthcare worker respondents interpreting the interviews as an evaluation of their individual performance in the clinics (especially as this study evaluates the NCD service), briefings will be made with all clinic health care workers explaining that: an independent researcher from LSHTM will perform the majority of the interviews; data will anonymised before reporting; and gaps identified will be addressed in a general training session for all staff. This will be reiterated in the written information sheet and verbal information provided.

## 4.6 Data management and protection

All paper data containing identifying information (e.g. signed consent forms) and those that do not contain identifying information (e.g. filled adherence questionnaires) will be held securely in locked filing cabinets at the MSF office in Irbid. Electronic data will be password protected, and held on password-protected computers with only identified team members given access to the data and password. The encoded cohort dataset will have the coding removed after final analysis for this evaluation is completed. All data (other than active service data) will be stored for ten years following completions of the project and will then be permanently deleted and destroyed. Responsibility for this rests with the principle investigator.

## 4.7 Confidentiality

All cohort data extracted from the programme database for analysis, survey data and qualitative data will be treated confidentiality with no names recorded in datasets. The semi-structured interviews will be conducted in a private space. The focus group discussions content will be treated as confidential and all participants will be requested to agree and adhere to the confidential nature of the discussions.

## 4.8 Independent review

This study protocol will be submitted to the Ethics Review Board instituted by MSF. Written authorisations to implement the study will also be obtained from the Ministry of Health in Irbid.

# 5. Study implementation

## 5.1 Collaborative partnership

This will be a collaborative partnership between MSF-OCA, the Jordanian Ministry of Health and the London School of Hygiene and Tropical Medicine.

## 5.2 Timeline

The proposed timeline is shown below. Month 1 is February 2017.

**Table 3.** Proposed timeline for MSF OCA Irbid NCD Programme Evaluation 2017

|  |  | | Month 2017-2018 | | | | | | | | | | | | |
| --- | --- | --- | --- | --- | --- | --- | --- | --- | --- | --- | --- | --- | --- | --- | --- |
|  | 1  F | 2  M | | 3 A | 4 M | 5  J | 6  J | 7  A | 8  S | 9  O | 10  N | 11  D | 12  J | 13  F |  |
| Finalise protocol |  |  | |  |  |  |  |  |  |  |  |  |  |  |  |
| Ethics approval |  |  | |  |  |  |  |  |  |  |  |  |  |  |  |
| Implementation |  |  | |  |  |  |  |  |  |  |  |  |  |  |  |
| Cohort routine data collection |  |  | |  |  |  |  |  |  |  |  |  |  |  |  |
| Adherence Survey |  |  | |  |  |  |  |  |  |  |  |  |  |  |  |
| Qualitative research |  |  | |  |  |  |  |  |  |  |  |  |  |  |  |
| Clinical audit |  |  | |  |  |  |  |  |  |  |  |  |  |  |  |
| Cost data collection |  |  | |  |  |  |  |  |  |  |  |  |  |  |  |
| Cross-sectional survey secondary analysis |  |  | |  |  |  |  |  |  |  |  |  |  |  |  |
| Cohort data analysis |  |  | |  |  |  |  |  |  |  |  |  |  |  |  |
| Qualitative data analysis |  |  | |  |  |  |  |  |  |  |  |  |  |  |  |
| Cost data analysis |  |  | |  |  |  |  |  |  |  |  |  |  |  |  |
| Dissemination |  |  | |  |  |  |  |  |  |  |  |  |  |  |  |
| Academic Papers |  |  | |  |  |  |  |  |  |  |  |  |  |  |  |

## 5.3 Dissemination plan

Ongoing data from the cohort study will be shared with local, national and international stakeholders every 6 months in order to inform and improve the delivery of NCD care by MSF and other public health agencies in the study area. Outputs from the rest of the study such as the qualitative research will include briefing papers, reports and peer-reviewed academic publications and presentations at key conferences and workshops.

Specific outputs will include a briefing paper and overview report of all findings for MSF-OCA. Peer-reviewed academic publications will include a paper based on the each of the core methodologies, i.e. cohort analysis and qualitative data analysis, with additional papers focusing on relevant, identified mental health and NCD comorbidities and on implementation of the NCD guideline.

All team members will be authors on the journal publications; with author ordering based on time inputs in to the study and designated roles.

## 5.4 Financial resources

Routine data cleaning and will be covered by the MSF programme budget. All other costs relating to the study, including travel, transcription and translation of qualitative data, will be covered by the Manson Unit (MSF-OCA).

# 6 Limitations

The study design does not allow us to ascertain clinical impact or effectiveness of the programme since we have not included a comparator group. In addition, the two-year time frame and cohort size involved in this evaluation mean that typical clinical endpoints used in the study of chronic conditions, such as all-cause mortality, fatal and non-fatal myocardial infarction, cannot be measured. It is well established that improvement in surrogate measures i.e. clinical or biochemical parameters such as blood pressure, cholesterol or HbA1c, represents a reduction in cardiovascular risk^[[1]](#footnote-1)^. If maintained, this would result in a reduction in morbidity and mortality. In addition, improvements in clinical parameters are assumed to be due to the intervention itself but may be as a result of other, indeterminate factors, such as a patient receiving NCD care and/or medications from another source concurrently. Insight will be gained regarding this latter potential bias through focus groups and semi-structured interviews.

While literacy rates are quite high among Syrians (92% for men; 81 % women), we recognise this could still lead to potential bias in the adherence survey and also that literacy rates will vary by sub-group (e.g. based on gender, age, socio-economic background). To try and reduce this bias, a data collector will administer the adherence questionnaire to patients with limited literacy (and this will be recorded on the questionnaire).

In addition, this is a single-country case study focusing on a specific population (Syrian refugees and vulnerable Jordanians living in north Jordan), whose findings may not be generalizable to other settings and populations. However, the study addresses a major research gap and its findings are likely to inform actors with an interest in NCDs in humanitarian settings. The focus groups and interviews will be conducted by staff members from the implementing organisation (MSF) and are response-based, increasing the risk of social desirability bias. These risks will be mitigated by comprehensive explanation of the study to participants, with emphasis that participation will not influence the care they receive, and supervision by the principal investigator. Purposive sampling of interviewees may introduce selection bias. To minimise this bias, a broad sample of patients with a variety of demographic characteristics and medical conditions will be selected. Quantitative data are subject to measurement error and rigorous data cleaning will be undertaken.

# 7 References

1. WHO | Global status report on noncommunicable diseases 2014. *WHO*. 2015.

2. *WHO | Noncommunicable Diseases Country Profiles 2011*. Geneva: World Health Organization; 2011. http://www.who.int/nmh/countries/2011/en/. Accessed April 23, 2016.

3. Health Service Utilization among Syrian Refugees with Chronic Health Conditions in Jordan. *PLoS One*. 2106;11(4):e0150088. doi:10.1371/journal.pone.0150088.

4. UNHCR. Syria Regional Refugee Response - Jordan - Inter-agency Information Sharing Portal. http://data.unhcr.org/syrianrefugees/country.php?id=107. Accessed December 16, 2016.

5. IFRC. *World Disasters Report 2012: Focus on Forced Migration and Displacement.* Geneva; 2012. http://www.ifrcmedia.org/assets/pages/wdr2012/.

6. UNHCR. Syria Regional Refugee Response - Regional Overview. http://data.unhcr.org/syrianrefugees/regional.php. Accessed April 23, 2016.

7. Barnett K, Mercer SW, Norbury M, et al. Epidemiology of multimorbidity and implications for health care, research, and medical education: a cross-sectional study. *Lancet*. 2012;380(9836):37-43. doi:10.1016/S0140-6736(12)60240-2.

8. Prince MJ, Wu F, Guo Y, et al. The burden of disease in older people and implications for health policy and practice. *Lancet*. 2015;385(9967):549-562. doi:10.1016/S0140-6736(14)61347-7.

9. Bierman AS, Tinetti ME, Prince M, et al. Precision medicine to precision care: managing multimorbidity. *Lancet*. 2016;388(10061):2721-2723. doi:10.1016/S0140-6736(16)32232-2.

10. *WHO | Global Status Report on Noncommunicable Diseases 2014*. Geneva; 2014. http://www.who.int/nmh/publications/ncd-status-report-2014/en/. Accessed April 23, 2016.

11. Barnett K, Mercer SW, Norbury M, et al. Epidemiology of multimorbidity and implications for health care, research, and medical education: a cross-sectional study. *Lancet (London, England)*. 2012;380(9836):37-43. doi:10.1016/S0140-6736(12)60240-2.

12. Gunn JM, Ayton DR, Densley K, et al. The association between chronic illness, multimorbidity and depressive symptoms in an Australian primary care cohort. *Soc Psychiatry Psychiatr Epidemiol*. 2012;47(2):175-184. doi:10.1007/s00127-010-0330-z.

13. Moussavi S, Chatterji S, Verdes E, et al. Depression, chronic diseases, and decrements in health: results from the World Health Surveys. *Lancet (London, England)*. 2007;370(9590):851-858. doi:10.1016/S0140-6736(07)61415-9.

14. *WHO | Global Action Plan for the Prevention and Control of NCDs 2013-2020*. Geneva; 2013. http://www.who.int/nmh/events/ncd_action_plan/en/. Accessed May 2, 2016.

15. Maher D, Harries AD, Zachariah R, Enarson D. A global framework for action to improve the primary care response to chronic non-communicable diseases: a solution to a neglected problem. *BMC Public Health*. 2009;9(1):355. doi:10.1186/1471-2458-9-355.

16. Miranda JJ, Kinra S, Casas JP, Davey Smith G, Ebrahim S. Non-communicable diseases in low- and middle-income countries: context, determinants and health policy. *Trop Med Int Health*. 2008;13(10):1225-1234. doi:10.1111/j.1365-3156.2008.02116.x.

17. Ebrahim S, Pearce N, Smeeth L, Casas JP, Jaffar S, Piot P. Tackling non-communicable diseases in low- and middle-income countries: is the evidence from high-income countries all we need? *PLoS Med*. 2013;10(1):e1001377. doi:10.1371/journal.pmed.1001377.

18. UNICEF Jordan - Media centre - Jordan Population and Housing Census 2015. https://www.unicef.org/jordan/media_10894.html. Accessed December 21, 2016.

19. United Nationa High Commissoner for Human Rights (UNHCR). Guide to UNHCR Supported Health Care Services in Jordan. 2013. available: http://data.unhcr.org/syrianrefugees/download.php?id=2862.

20. *Hashemite Kingdom of Jordan Syrian Crisis. Health Needs Assessment. March 2014 - Report to the Ministry of Health. Premiere Urgence - Aide Medicale Internationale.*; 2014. https://data.unhcr.org/syrianrefugees/download.php?id=6472.

21. Doocy S, Lyles E, Akhu-Zaheya L, Oweis A, Al Ward N, Burton A. Health Service Utilization among Syrian Refugees with Chronic Health Conditions in Jordan. Wang Y, ed. *PLoS One*. 2016;11(4):e0150088. doi:10.1371/journal.pone.0150088.

22. EMRO Health System Profile-Jordan. Regional Health Systems Observatory. World Health Organization. 2006. http://apps.who.int/medicinedocs/documents/s17296e/s17296e.pdf. Accessed May 2, 2016.

23. UNHCR. *Health Sector Jordan Monthly Report 15 December 2014*.; 2014. https://data.unhcr.org/syrianrefugees/download.php?id=7762. Accessed December 20, 2016.

24. Doocy S, Lyles E, Roberton T, Akhu-Zaheya L, Oweis A, Burnham G. Prevalence and care-seeking for chronic diseases among Syrian refugees in Jordan. *BMC Public Health*. 2015;15:1097. doi:10.1186/s12889-015-2429-3.

25. Nguyen T-M-U, La Caze A, Cottrell N, Nguyen BPharm T-M-U. What are validated self-report adherence scales really measuring?: a systematic review. doi:10.1111/bcp.12194.

26. Horne R, Weinman J. Patients’ beliefs about prescribed medicines and their role in adherence to treatment in chronic physical illness. *J Psychosom Res*. 1999;47(6):555-567. doi:10.1016/S0022-3999(99)00057-4.

27. Alsous M, Alhalaiqa F, Abu Farha R, Abdel Jalil M, McElnay J, Horne R. Reliability and validity of Arabic translation of Medication Adherence Report Scale (MARS) and Beliefs about Medication Questionnaire (BMQ)-specific for use in children and their parents. Choonara I, ed. *PLoS One*. 2017;12(2):e0171863. doi:10.1371/journal.pone.0171863.

28. Horne R, Weinman J, Hankins M. The beliefs about medicines questionnaire: The development and evaluation of a new method for assessing the cognitive representation of medication. *Psychol Health*. 1999;14(1):1-24. doi:10.1080/08870449908407311.

29. UNHCR; UNFPA; IMC-Jordan. *Population Based Health Access Assessment for Syrian Refugees in Non-Camp Settings Throughout Jordan: With Sub-Investigation on Non-Communicable Disease Management*. Amman; 2014. http://reliefweb.int/report/jordan/population-based-health-access-assessment-syrian-refugees-non-camp-settings-throughout.

30. De Silva MJ, Rathod SD, Hanlon C, et al. Evaluation of district mental healthcare plans: the PRIME consortium methodology. *Br J Psychiatry*. 2016;(Suppl 56):s63-70. doi:10.1192/bjp.bp.114.153858.

# Annex 1: Consent Forms

## Annex 1a: Consent statement for patients of MSF NCD service for the qualitative research (focus group discussions)

This informed consent form is for patients of the MSF Non-communicable Disease (NCD) service in Irbid governorate, Jordan, whom we are inviting to participate in an evaluation of the service, entitled “*Evaluation of a primary care-based NCD service in Irbid, Jordan.”*

**Principal Investigator**: Dr Éimhín Ansbro

**Organisation:** London School of Hygiene and Tropical Medicine, Médecins sans Frontières, Ministry of Health, Hashemite Kingdom of Jordan.

**Study Sponsor:** MSF

**Project:** Evaluation of a primary care-based NCD service in Irbid, Jordan

**This Informed Consent Form has two parts:**

- Part 1: Information Sheet (to share information about the study with you)
- Part 2: Certificate of Consent (for signatures if you choose to participate)

**You will be given a copy of the full Informed Consent Form**

**Part 1: Information Sheet**

Before collecting any information from the participants, administer the following consent statement and ask for written consent.

***Introduction***

*I am (name), working for MSF as (role). I am part of the research team evaluating the Non-communicable Disease (NCD) service provided by MSF in Irbid. This service provides treatment for certain chronic illnesses, such as diabetes, high blood pressure and asthma. I am going to give you information and invite you to be part of this research. You do not have to decide today whether or not you will participate in the research. Before you decide, you can talk to anyone you feel comfortable with about the research. In this form, there may be some words that you do not understand. Please ask me to stop as we go through the information and I will take time to explain. If you have questions later, please ask me or another member of staff.*

***Purpose of the study***

*In order to evaluate our NCD service in Irbid we would like to ask you some questions about your experience of managing your condition, accessing care, and the care you’ve received here in the clinic. This will involve your participation in a group discussion that will take about one and a half hours and may be followed by an individual interview that will take about one hour (on another day). . We will share the findings with the patients of the service and will then use the findings to help with evaluating and improving the NCD services, as well as sharing them more broadly through conference presentations and scientific publication by MSF.*

***Participant Selection***

*We have asked you to participate because we feel your experience as a patient of the service can contribute to our understanding of how the service is working currently.*

***Risks and benefits of participation***

*Participating in the discussion group is entirely voluntary. It is your choice whether to participate or not. While taking part will not directly benefit you in any way, your participation will help us to find out how the NCD service is working, to potentially improve it and to apply what we have learned in other communities. Refusal to participate will not harm you in any way, and will NOT affect the medical treatment that you will receive.*

***Right to withdraw consent to participate***

*You can also withdraw your consent at any time before OR during the discussion, or afterwards until the study is complete, by contacting the MSF clinic staff (Hashim Taani: irbid-liaison@oca.msf.org). In this event, your data will be withdrawn from the database (however, once data collection is complete and analysis is underway, it is no longer possible to withdraw data.) We would also ask your permission to contact you after the focus group discussion to ask you to participate in an individual interview in the next few weeks. The research coordinator will record the best way to contact you. You can withdraw your permission to contact you at any time (by contacting our the research team) and you can also refuse to participate in the individual interviews without any represcussions.*

***Focus group discussion process and content***

*We are asking you to take part in a group discussion with 7-8 other people who have had similar experiences. The discussion or interview will take place in the MSF clinic, and no one else but the people who take part in the discussion and guide or myself will be present during this discussion. It will take about one and a half hours.*

*The group discussion leader, (name), will guide this discussion. The group discussion will start with (name) making sure that you are comfortable. We can also answer questions about the research that you might have. Then we will ask you questions about your NCD condition, your experience of accessing and attending the MSF service, any benefits or difficulties in accessing or attending the service, your experience of managing your condition, including taking medications and making lifestyle changes and the types of support available or that you would like to have. We will not ask you to share personal beliefs, practices or stories and you do not have to share any knowledge that you are not comfortable sharing.*

***Confidentiality and anonymity***

*We will ask you and others in the group not to talk to people outside the group about what was said in the group. We will, in other words, ask each of you to keep what was said in the group confidential. You should know, however, that we cannot stop or prevent participants who were in the group from sharing things that should be confidential. Please also be careful not to share information about yourself, such as about a particular illness or symptoms that you wish to keep private.*

*All the findings from this discussion will be confidential and anonymous. The discussion will be audio recorded and participants will not be identified by name on the recording. The recordings will only be heard by the research team and translator. All the transcribed written records of the recording will be kept privately and anonymously so that no one can link anything you say in the discussion group back to you. The recording and written transcriptions will be securely stored and only accessible by approved study team members. In the future, the anonymised data (without any identifying information about you) may be made available to other researchers in a public data store.*

*There is a risk that you may share some personal or confidential information by chance, or that you may feel uncomfortable talking about some of the topics. However, we do not wish for this to happen. You do not have to answer any question or take part in the discussion and you can stop at any time if you feel the question(s) are too personal or if talking about them makes you uncomfortable.*

***Study approvals***

*MSF and the Ministry of Health in Jordan have given their approval for the interviews to be conducted.*

***Summary***

*To confirm, you do not have to take part in this research if you do not wish to, and choosing to participate will not affect your medical treatment in any way. You may stop participating in the interview at any time without your medical care being affected. I will give you an opportunity at the end of the discussion to review your remarks, and you can ask to modify or remove portions of those, if you do not agree with my notes or if I did not understand you correctly.*

*If you have any questions, you can ask them now or later, you may contact any of the following:*

**Study Contact Details**

| **Name** | **Position** | **Phone Number** | **Email address** |
| --- | --- | --- | --- |
| Dr Éimhín Ansbro | Principal Investigator, LSHTM |  | eimhin.ansbro@lshtm.ac.uk |
| Dr. Majed Asad | Head of NCD Directorate, Ministry of Health | +962 79 574 0531 | [majedasad@yahoo.com](mailto:majedasad@yahoo.com) |
| Hashim Taani | Research Coordinator, MSF, Irbid | +962 79 749 6191 | [irbid-liaison@oca](mailto:irbid-liaison@oca.msf.oca) |

P**art 2: Certificate of Consent (patient focus groups)**

*I agree to participate in a research study evaluating the MSF NCD programme in Irbid, Jordan, I have read the information provided above, or it has been read to me. I have had the opportunity to ask questions about it and any questions that I have asked have been answered to my satisfaction. I am aware that I can withdraw my consent (including consent to be re-contacted) by contacting the research coordinator and these details have been provided to me.*

Respondent number: ______________ Date:_____/_____/_________(Day/month/year)

I, ____________________________________________________________ _(Print patient’s name/guardian)

consent voluntarily to: (Tick the box(es) that apply)

a) ☐ Participate in a focus group discussion

b) ☐Be audio-recorded during the focus group discussion

c) ☐Be re-contacted by the research team to be invited to participate in an individual interview

__________________________________________________________ (Signature)

Designation, i.e. Patient/parent/guardian: ____________________(Print)

**If illiterate**

A literate witness must sign (if possible, this person should be selected by the patient and should have no connection to the research team).

*I have witnessed the accurate reading of the consent form to the potential participant, and the individual has had the opportunity to ask questions. I confirm that the individual has given consent freely.*

Print name of witness________________________________ Thumbprint of participant:

Signature of witness ________________________________

Date ________________________ (Day/month/year)

***Statement by the researcher/person taking consent***

*I have accurately read out the information sheet to the potential participant, and to the best of my ability made sure that the participant understands that the following will be done:*

*1. Participation is voluntary and can be withdrawn at any time. Refusal to participate will not affect medical care in anyway.*

*2. Data may be withdrawn up to a certain point in the analysis, will be anonymised and is only accessible to the research team*

*3. Study findings will be shared with the participants, MoH, MSF and broader global health community through publication.*

*I confirm that the participant was given an opportunity to ask questions about the study, and all the questions asked by the participant have been answered correctly and to the best of my ability. I confirm that the individual has not been coerced into giving consent, and the consent has been given freely and voluntarily.*

*A copy of this ICF has been provided to the participant.*

*Print Name of Researcher**/person taking the consent________________________________________*

*Signature of Researcher /person taking the consent_________________________________________*

*Date ____________________________________________ (Day/month/year)*

## Annex 1b: Consent statement for patients of MSF NCD service for the qualitative research (semi-structured interviews)

This informed consent form is for patients of the MSF Non-communicable Disease (NCD) service in Irbid governorate, Jordan, whom we are inviting to participate in an evaluation of the service, entitled “*Evaluation of a primary care-based NCD service in Irbid, Jordan.”*

**Principal Investigator**: Dr Éimhín Ansbro

**Organisation:** London School of Hygiene and Tropical Medicine, Médecins sans Frontières, Ministry of Health, Hashemite Kingdom of Jordan.

**Study Sponsor:** MSF

**Project:** Evaluation of a primary care-based NCD service in Irbid, Jordan

**This Informed Consent Form has two parts:**

- - Part 1: Information Sheet (to share information about the study with you)
  - Part 2: Certificate of Consent (for signatures if you choose to participate)

**You will be given a copy of the full Informed Consent Form**

**Part 1: Information Sheet**

Before collecting any information from the participants, administer the following consent statement and ask for written consent.

***Introduction***

*I am (name), working for MSF as (role). I am part of the research team evaluating the Non-communicable Disease (NCD) service provided by MSF in Irbid. This service provides treatment for certain chronic illnesses, such as diabetes, high blood pressure and asthma. I am going to give you information and invite you to be part of this research. You do not have to decide today whether or not you will participate in the research. Before you decide, you can talk to anyone you feel comfortable with about the research. In this form, there may be some words that you do not understand. Please ask me to stop as we go through the information and I will take time to explain. If you have questions later, please ask me or another member of staff.*

***Purpose of the study***

*In order to evaluate our NCD service in Irbid we would like to ask you some questions about your experience of managing your condition, accessing care, and the care you’ve received here in the clinic. This will take about one hour. We will share the findings with the patients of the service and will then use the findings to help with evaluating and improving the NCD services, as well as sharing them more broadly through conference presentations and scientific publication by MSF.*

***Participant Selection***

*We have asked you to participate because we feel your experience as a patient of the service can contribute to our understanding of how the service is working currently.*

***Risks and benefits of participation***

*Participating in the interview is entirely voluntary. It is your choice whether to participate or not. While taking part will not directly benefit you in any way, your participation will help us to find out how the NCD service is working, to potentially improve it and to apply what we have learned in other communities. Refusal to participate will not harm you in any way, and will NOT affect the medical treatment that you will receive.*

***Right to withdraw consent to participate***

*You can also withdraw your consent at any time before OR during the interview, or afterwards until the study is complete, by contacting the MSF clinic staff (Hashim Taani: irbid-liaison@oca.msf.org). In this event, your data will be withdrawn from the database. (However, once data collection is complete and analysis is underway, it is no longer possible to withdraw data.)*

***Interview process and content***

*During the interview, (name), the research coordinator, will sit down with you in a comfortable and private place at the clinic. If you do not wish to answer any of the questions during the interview, you may say so and the interviewer will move on to the next question. No one else but the interviewer will be present unless you would like someone else to be there.*

***Confidentiality and anonymity***

*The discussion will be audio recorded and you will not be identified by name on the recording. The recordings will only be heard by the research team and translator. All the transcribed written records of the recording will be kept privately and anonymously so that no one can link anything you say in the interview back to you. The recording and written transcriptions will be securely stored and only accessible by approved study team members. In the future, the anonymised data (without any identifying information about you) may be made available to other researchers in a public data store.*

*There is a risk that you may share some personal or confidential information by chance, or that you may feel uncomfortable talking about some of the topics. However, we do not wish for this to happen. You do not have to answer any question or take part in the interview if you feel the question(s) are too personal or if talking about them makes you uncomfortable.*

***Study approvals***

*MSF and the Ministry of Health in Jordan have given their approval for the interviews to be conducted.*

***Summary***

*To confirm, you do not have to take part in this research if you do not wish to do so, and choosing to participate will not affect your medical treatment in any way. You may stop participating in the interview at any time that you wish without your medical care being affected. I will give you an opportunity at the end of the interview/discussion to review your remarks, and you can ask to modify or remove portions of those, if you do not agree with my notes or if I did not understand you correctly If you have any questions, you can ask them now or later, you may contact any of the following:*

**Study Contact Details**

| **Name** | **Position** | **Phone Number** | **Email address** |
| --- | --- | --- | --- |
| Dr Éimhín Ansbro | Principal Investigator, LSHTM |  | eimhin.ansbro@lshtm.ac.uk |
| Dr. Majed Asad | Head of NCD Directorate, Ministry of Health | +962 79 574 0531 | [majedasad@yahoo.com](mailto:majedasad@yahoo.com) |
| Hashim Taani | Research Coordinator, MSF, Irbid | +962 79 749 6191 | [irbid-liaison@oca](mailto:irbid-liaison@oca.msf.oca) |

**Part 2: Certificate of Consent (patient interviews)**

*I agree to participate in a research study evaluating the MSF NCD programme in Irbid, Jordan. I have read the information provided above, or it has been read to me. I have had the opportunity to ask questions about it and any questions that I have asked have been answered to my satisfaction. I am aware that I can withdraw my consent by contacting the research coordinator and these details have been provided to me.*

Respondent number: ______________ Date: ­­­­­­­­­­­_____________________ (Day/month/year)

I, ______________________________________________________ (Print patient’s or guardian’s name)

consent voluntarily to: (Tick the box(es) that apply)

a) ☐ participate in an individual interview

b) ☐ be audio-recorded during the interview

____________________________________ ___________________ (Signature)

Designation: _______________________________________ (Print i.e. Patient/parent/guardian)

**If illiterate**

A literate witness must sign (if possible, this person should be selected by the patient and should have no connection to the research team).

*I have witnessed the accurate reading of the consent form to the potential participant, and the individual has had the opportunity to ask questions. I confirm that the individual has given consent freely.*

Print name of witness_____________________________ Thumbprint of participant:

Signature of witness ________________________________

Date ________________________ (Day/month/year)

***Statement by the researcher/person taking consent***

*I have accurately read out the information sheet to the potential participant, and to the best of my ability made sure that the participant understands that the following will be done:*

*1. Participation is voluntary and can be withdrawn at any time. Refusal to participate will not affect medical care in anyway.*

*2. Data may be withdrawn up to a certain point in the analysis, will be anonymised and is only accessible to the research team*

*3. Study findings will be shared with the participants, MoH, MSF and broader global health community through publication.*

*I confirm that the participant was given an opportunity to ask questions about the study, and all the questions asked by the participant have been answered correctly and to the best of my ability. I confirm that the individual has not been coerced into giving consent, and the consent has been given freely and voluntarily.*

**A copy of this ICF has been provided to the participant.**

Print Name of Researcher/person taking the consent________________________________________

Signature of Researcher /person taking the consent_________________________________________

Date ___________________________ (Day/month/year)

## Annex 1c: Consent statement for patients of MSF NCD service for the medication adherence questionnaire

This informed consent form is for patients of the MSF Non-communicable Disease (NCD) service in Irbid governorate, Jordan, whom we are inviting to participate in an evaluation of the service, entitled “Evaluation of a primary care-based NCD service in Irbid, Jordan.”

**Principal Investigator:**Dr Éimhín Ansbro

**Organisation**: London School of Hygiene and Tropical Medicine, Médecins sans Frontières, Ministry of Health, Hashemite Kingdom of Jordan.

**Study Sponsor:** MSF

**Project:** Evaluation of a primary care-based NCD service in Irbid, Jordan

**This Informed Consent Form has two parts:**

- - Part 1: Information Sheet (to share information about the study with you)
  - Part 2: Certificate of Consent (for signatures if you choose to participate)

**You will be given a copy of the full Informed Consent Form**

**Part 1: Information Sheet**

Before collecting any information from the participants, administer the following consent statement and ask for written consent.

***Introduction***

*I am (name), working for MSF as (role). I am part of the research team evaluating the Non-communicable Disease (NCD) service provided by MSF in Irbid. This service provides treatment for certain chronic illnesses, such as diabetes, high blood pressure and asthma. I am going to give you information and invite you to be part of this research. You do not have to decide today whether or not you will participate in the research. Before you decide, you can talk to anyone you feel comfortable with about the research. In this form, there may be some words that you do not understand. Please ask me to stop as we go through the information and I will take time to explain. If you have questions later, please ask me or another member of staff.*

**Purpose of the study**

*In order to evaluate our NCD service in Irbid we would like to ask you some questions about your experience of taking medications to manage your condition. This will involve filling out a quick survey that should take less than ten minutes. We will share the findings with patients of the service and will then use the findings to help with evaluating and improving the NCD services, as well as sharing them more broadly through conference presentations and scientific publication by MSF.*

**Participant Selection**

*We have asked you to participate because we feel your experience as a patient of the service can contribute to our understanding of how the service is working currently.*

**Voluntary consent to participate and right to withdraw**

*Participating in the survey is entirely voluntary. You can also withdraw your consent at any time before OR during the survey, or afterwards until the study is complete, by contacting the MSF clinic staff (Hashim Taani: irbid-liaison@oca.msf.org). In this event, your data will be withdrawn from the database (however, once data collection is complete and analysis is underway, it is no longer possible to withdraw data.)*

***Interview process and content***

*During the interview, (name), the research coordinator, will sit down with you in a comfortable and private place at the clinic. If you do not wish to answer any of the questions during the interview, you may say so and the interviewer will move on to the next question. No one else but the interviewer will be present unless you would like someone else to be there.*

**Risks and benefits of participation**

*It is your choice whether to participate or not. While taking part will not directly benefit you in any way, your participation will help us to understand how the NCD service is working, to potentially improve it and to apply what we have learned in other communities. Refusal to participate will not harm you in any way, and will NOT affect the medical treatment that you will receive. You can also withdraw your consent at any time before OR during the survey, or afterwards until the study is complete, by contacting the MSF clinic staff (Hashim Taani: irbid-liaison@oca.msf.org). In this event, your data will be withdrawn from the database (however, once data collection is complete and analysis is underway, it is no longer possible to withdraw data.)*

**Survey process and content**

*We are asking you to fill out a short questionnaire here in the clinic, which should take less than ten minutes. The research assistant will sit down with you in a comfortable and private place at the clinic. You can read and fill the questionnaire yourself or if you have any difficulty the researcher can help you. The survey contains questions about how you personally take your medications. We want to understand how people manage their medications in “real life”. There are no right or wrong answers.*

**Confidentiality and anonymity**

*The survey will not record any names or other identifying information about you. The paper and computerised records will be securely stored and only accessible by approved study team members. In the future, the anonymised data (without any identifying information about you) may be made available to other researchers in a public data store.*

**Study Approvals**

*MSF and the Ministry of Health in Jordan have given their approval for this survey to be conducted.*

**Summary**

*To confirm, you do not have to take part in this research if you do not wish to do so, and choosing to participate will not affect your medical treatment in any way. You may stop participating in the survey at any time that you wish without your medical care being affected. If you have any questions, you can ask them now or later.*

*If you have any questions later, you may contact any of the following:*

**Study Contact Details**

| **Name** | **Position** | **Phone Number** | **Email address** |
| --- | --- | --- | --- |
| Dr Éimhín Ansbro | Principal Investigator, LSHTM |  | eimhin.ansbro@lshtm.ac.uk |
| Dr. Majed Asad | Head of NCD Directorate, Ministry of Health | +962 79 574 0531 | [majedasad@yahoo.com](mailto:majedasad@yahoo.com) |
| Hashim Taani | Research Coordinator, MSF, Irbid | +962 79 749 6191 | [irbid-liaison@oca](mailto:irbid-liaison@oca.msf.oca) |

**Part 2: Certificate of Consent (patient adherence questionnaire)**

*I agree to participate in a research study evaluating the MSF NCD programme in Irbid, Jordan. I have read the information provided above, or it has been read to me. I have had the opportunity to ask questions about it and any questions that I have asked have been answered to my satisfaction.*

Respondent number: ______________ Date: ­­­­­­­­­­­_____________________ (Day/month/year)

I, ______________________________________________________ consent voluntarily. (Print patient’s or guardian’s name)

____________________________________ ___________________ (Signature)

Designation: _______________________________________ (Print i.e. Patient/parent/guardian)

**If illiterate:** A literate witness must sign (if possible, this person should be selected by the patient and should have no connection to the research team).

*I have witnessed the accurate reading of the consent form to the potential participant, and the individual has had the opportunity to ask questions. I confirm that the individual has given consent freely.*

Print name of witness________________________________ Thumbprint of participant:

Signature of witness ________________________________

Date ________________________ (Day/month/year)

**Statement by the researcher/person taking consent**

*I have accurately read out the information sheet to the potential*

*participant, and to the best of my ability made sure that the participant understands:*

*1. Participation is voluntary and can be withdrawn at any time. Refusal to participate will not affect medical care in anyway.*

*2. Data may be withdrawn up to a certain point in the analysis, will be anonymised and is only accessible to the research team.*

*3. Study findings will be shared with the participants, MoH, MSF and broader global health community through publication.*

*I confirm that the participant was given an opportunity to ask questions about the study, and all the questions asked by the participant have been answered correctly and to the best of my ability. I confirm that the individual has not been coerced into giving consent, and the consent has been given freely and voluntarily.*

**A copy of this ICF has been provided to the participant.**

Print Name of Researcher/person taking the consent________________________

Signature of Researcher /person taking the consent__________________________

Date _________________________(Day/month/year)

## Annex 1d. Consent statement for health staff for the qualitative research

This informed consent form is for current or former staff of the MSF Non-communicable disease (NCD )service in Irbid governorate, Jordan, whom we are inviting to participate in an evaluation of the service, entitled “*Evaluation of a primary care-based NCD service in Irbid, Jordan.”*

**Principal Investigator:** Dr Éimhín Ansbro

**Organisation:** London School of Hygiene and Tropical Medicine, Médecins sans Frontières, Ministry of Health, Hashemite Kingdom of Jordan.

**Study Sponsor:** MSF

**Project:** Evaluation of a primary care-based NCD service in Irbid, Jordan

**This Informed Consent Form has two parts:**

• Part 1: Information Sheet (to share information about the study with you)

• Part 2: Certificate of Consent (for signatures if you choose to participate)

**You will be given a copy of the full Informed Consent Form**

**Part 1: Information Sheet**

Before collecting any information from the expert participants, administer the following consent statement and ask for written consent.

**Introduction**

*I am Éimhin Ansbro, a researcher working with MSF and the London School of Hygiene and Tropical Medicine. I am part of the research team evaluating the Non-communicable Disease (NCD) service provided by MSF in Irbid. This service provides treatment for certain chronic illnesses, such as diabetes, high blood pressure and asthma. I am going to give you information and invite you to be part of this research. You do not have to decide today whether or not you will participate. Before you decide, you can talk to anyone you feel comfortable with about the research. Please ask me to stop as we go through the information if you wish me to clarify any detail. If you have questions later, please ask me or another member of staff.*

**Purpose of the study**

*In order to evaluate our programme of NCD services in Irbid we would like to ask you some questions about your experience of supporting NCD care in Irbid. We will use the findings to help with evaluating and improving the NCD services, research and scientific publication by MSF. Please note that this is not an evaluation of your individual performance and will have no bearing on your employment with MSF now or in the future. We are only interested in the programme overall.*

**Participant Selection**

*You have been selected for interview because your experience of the programme will contribute significantly to our understanding of the current service. We have asked a range of health care and workers and management staff who are currently or were formerly involved with the Irbid NCD service to gain the broadest view of the service.*

**Interview process and content**

*A one-to-one interview will be conducted by myself or the local research coordinator either in person in the MSF office in Irbid or over Skype. It will last about one hour. It will cover subjects like patient access to NCD care, sources of information and support for NCD patients and staff, challenges and benefits of the NCD programme for patients and staff.*

**Voluntary participation and right to withdraw**

*Participating in the discussion is entirely voluntary. You do not have to answer any question or take part in the interview if you feel the question(s) are too personal or if talking about them makes you uncomfortable. You can also withdraw your consent at any time before OR during the discussion, or afterwards until the study is complete, by contacting the MSF clinic staff (Hashim Taani: irbid-liaison@oca.msf.org). In this event, your data will be withdrawn from the database. (However, once data collection is complete and analysis is underway, it is no longer possible to withdraw data.)*

**Risks and benefits of participation**

*Taking part will not directly benefit you in any way but the study findings will benefit the NCD programme users and staff in general and will be shared with the Jordanian MoH, MSF and the wider global health community through publication.*

*Refusal to participate will not harm you in any way. The choice that you make will have no bearing on your job or on any work-related evaluations or reports.*

**Confidentiality and Anonymity**

*All the findings from this discussion will be confidential and anonymous. The interviews will be audio recorded. The recordings will only be heard by the research team and translator. All the transcribed written records of the recording will be kept privately and anonymously so that no one can link anything you say in the interview back to you. The recording and written transcriptions will be securely stored and only accessible by approved study team members. In the future, the anonymised data (without any identifying information about you) may be made available to other researchers in a public data store.*

**Study Approvals**

*MSF and the Jordanian Ministry of Health have given their approval for these interviews to be conducted. If you have any questions, you can ask them now or later.*

*If you have any questions later, you may contact any of the following:*

**Study Contact Details**

| **Name** | **Position** | **Phone Number** | **Email address** |
| --- | --- | --- | --- |
| Dr Éimhín Ansbro | Principal Investigator, LSHTM |  | eimhin.ansbro@lshtm.ac.uk |
| Dr. Majed Asad | Head of NCD Directorate, Ministry of Health | +962 79 574 0531 | [majedasad@yahoo.com](mailto:majedasad@yahoo.com) |
| Hashim Taani | Research Coordinator, MSF, Irbid | +962 79 749 6191 | [irbid-liaison@oca](mailto:irbid-liaison@oca.msf.oca) |

**Part 2: Certificate of Consent (health staff)**

*I agree to participate in a research study evaluating the MSF NCD programme in Irbid, Jordan. I have read this information. I have had the opportunity to ask questions about it and any questions that I have asked have been answered to my satisfaction.*

Respondent number: ______________ Date: ­­­­­­­­­­­_____________________ (Day/month/year)

I, ______________________________________________________ (Print name)

consent voluntarily to: (Tick the box(es) that apply)

a) ☐ Participate in an interview

b) ☐Be audio-recorded during the interview

____________________________________ ___________________ (Signature)

**Statement by the researcher/person taking consent**

*I have accurately read out the information sheet to the potential participant, and to the best of my ability made sure that the participant understands the following:*

1*. Participation is voluntary and can be withdrawn at any time. Refusal to participate will not affect medical care in anyway.*

*2. Data may be withdrawn up to a certain point in the analysis, will be anonymised and is only accessible to the research team*

*3. Study findings will be shared with the participants, MoH, MSF and broader global health community through publication.*

*I confirm that the participant was given an opportunity to ask questions about the study, and all the questions asked by the participant have been answered correctly and to the best of my ability. I confirm that the individual has not been coerced into giving consent, and the consent has been given freely and voluntarily.*

***A copy of this ICF has been provided to the participant.***

*Name of Researcher/person taking the consent_______________________________(Print)*

*_______________________________(Signature)*

*Date ___________________________ (Day/month/year)*

## Annex 1e. Consent statement for key stakeholders for the qualitative research

This informed consent form is for key stakeholders of the MSF Non-communicable Disease (NCD) service in Irbid governorate, Jordan, whom we are inviting to participate in an evaluation of the service, entitled “*Evaluation of a primary care-based NCD service in Irbid, Jordan.”*

**Principal Investigator:** Dr Éimhín Ansbro

**Organisation:** London School of Hygiene and Tropical Medicine, Médecins sans Frontières, Ministry of Health, Hashemite Kingdom of Jordan.

**Study Sponsor:** MSF

**Project:** Evaluation of a primary care-based NCD service in Irbid, Jordan

**This Informed Consent Form has two parts:**

• Part 1: Information Sheet (to share information about the study with you)

• Part 2: Certificate of Consent (for signatures if you choose to participate)

**You will be given a copy of the full Informed Consent Form**

**Part 1: Information Sheet**

Before collecting any information from the expert participants, administer the following consent statement and ask for written consent.

**Introduction**

*I am Éimhin Ansbro, a researcher working with MSF and the London School of Hygiene and Tropical Medicine. I am part of the research team evaluating the Non-communicable Disease (NCD) service provided by MSF in Irbid. This service provides treatment for certain chronic illnesses, such as diabetes, high blood pressure and asthma. I am going to give you information and invite you to be part of this research. You do not have to decide today whether or not you will participate in the research. Before you decide, you can talk to anyone you feel comfortable with about the research. Please ask me to stop as we go through the information if you wish me to clarify any detail. If you have questions later, please ask me or another member of staff.*

**Purpose of the study**

*In order to evaluate our programme of NCD services in Irbid we would like to ask you some questions about your experience of the MSF NCD service in Irbid. We will use the findings to help with evaluating and improving the NCD service in Irbid,, research and scientific publication by MSF.*

**Participant Selection**

*You have been selected for interview because of your knowledge and experience of healthcare in Jordan, particular NCD services and/or because you represent the community targeted by the MSF service. Your contribution will add significantly to our understanding of the current service in Irbid.*

**Interview process and content**

*Your participation will involve a one-to-one interview in person with one of the research team at the MSF office in Irbid or Ministry of Health locations. This will, last approximately one hour. It will cover subjects such as patient access to NCD care, sources of information and support for NCD patients, staff and community, challenges and benefits of the NCD programme for patients, staff, community and health service in Irbid.*

**Confidentiality and anonymity**

*All the findings from this discussion will be confidential and anonymous. The interviews will be audio recorded. The recordings will only be heard by the research team and translator. All the transcribed written records of the recording will be kept privately and anonymously so that no one can link anything you say in the interview back to you. The recording and written transcriptions will be securely stored and only accessible by approved study team members. . In the future, the anonymised data (without any identifying information about you) may be made available to other researchers in a public data store.*

**Risks and benefits of participation**

*Participating will not directly benefit you in any way but the study findings will benefit the NCD programme users and staff and will be shared with the Jordanian MoH, MSF and the wider global health community through publication.*

*Refusal to participate will not harm you in any way. You do not have to answer any question or take part in the interview if you feel the question(s) are too personal or if talking about them makes you uncomfortable.*

**Voluntary participation and right to withdraw**

*Participating in the interview is entirely voluntary. You can also withdraw your consent at any time before OR during the discussion, or afterwards until the study is complete, by contacting the MSF clinic staff (Hashim Taani: irbid-liaison@oca.msf.org). In this event, your data will be withdrawn from the database (however, once data collection is complete and analysis is underway, it is no longer possible to withdraw data.)*

**Study approvals**

*MSF and the Jordanian Ministry of Health have given their approval for these interviews to be conducted. If you have any questions, you can ask them now or later.*

*If you have any questions later, you may contact any of the following:*

**Study Contact Details**

| **Name** | **Position** | **Phone Number** | **Email address** |
| --- | --- | --- | --- |
| Dr Éimhín Ansbro | Principal Investigator, LSHTM |  | eimhin.ansbro@lshtm.ac.uk |
| Dr. Majed Asad | Head of NCD Directorate, Ministry of Health | +962 79 574 0531 | [majedasad@yahoo.com](mailto:majedasad@yahoo.com) |
| Hashim Taani | Research Coordinator, MSF, Irbid | +962 79 749 6191 | [irbid-liaison@oca](mailto:irbid-liaison@oca.msf.oca) |

**Part 2: Certificate of Consent (stakeholders)**

*I agree to participate in a research study evaluating the MSF NCD programme in Irbid, Jordan. I have read this information. I have had the opportunity to ask questions about it and any questions that I have asked have been answered to my satisfaction.*

Respondent number: ______________ Date: ­­­­­­­­­­­_____________________ (Day/month/year)

I, ______________________________________________________ (Print name)

consent voluntarily to: (Tick the box(es) that apply)

a) ☐ participate in an interview

b) ☐ be audio-recorded during the interview

____________________________________ ___________________ (Signature)

**Statement by the researcher/person taking consent**

*I have accurately read out the information sheet to the potential participant, and to the best of my ability made sure that the participant understands the following:*

1*. Participation is voluntary and can be withdrawn at any time. Refusal to participate will not affect medical care in anyway.*

*2. Data may be withdrawn up to a certain point in the analysis, will be anonymised and is only accessible to the research team*

*3. Study findings will be shared with the participants, MoH, MSF and broader global health community through publication.*

*I confirm that the participant was given an opportunity to ask questions about the study, and all the questions asked by the participant have been answered correctly and to the best of my ability. I confirm that the individual has not been coerced into giving consent, and the consent has been given freely and voluntarily.*

***A copy of this ICF has been provided to the participant.***

*Name of Researcher/person taking the consent_______________________________(Print)*

*_______________________________(Signature)*

*Date _________________________ (Day/month/year)*

## Annex 1f. Consent statement for observations of patient group counselling and clinical consultations

This is for informed verbal consent form for patients receiving Non-communicable Disease (NCD) services in Irbid governorate, Jordan, which is for an evaluation of the service, entitled “*Evaluation of a primary care-based NCD service in Irbid, Jordan.”*

**Principal Investigator:** Dr Éimhín Ansbro

**Organisation:** London School of Hygiene and Tropical Medicine, Médecins sans Frontières, Ministry of Health, Hashemite Kingdom of Jordan.

**Study Sponsor:** MSF

**Project:** Evaluation of a primary care-based NCD service in Irbid, Jordan

**This Informed Consent has two parts:**

• Part 1: Information Sheet (to share information about the study with you)

• Part 2: Certificate of Consent (signed by Éimhin Ansbro to confirm you have given verbal consent to be observed in group counselling and/or clinical consultations)

**You will be given a copy of the full Informed Consent Form**

**Part I: Information Sheet**

Before making any observations, administer the following consent statement and ask for verbal consent.

**Introduction**

*I am Éimhin Ansbro, a researcher working with MSF and the London School of Hygiene and Tropical Medicine. I am part of the research team evaluating the Non-communicable Disease (NCD) service provided by MSF in Irbid. This service provides treatment for certain chronic illnesses, such as diabetes, high blood pressure and asthma. I am going to give you information and invite you to be part of this research. You do not have to decide today whether or not you will participate in the research. Before you decide, you can talk to anyone you feel comfortable with about the research. Please ask me to stop as we go through the information if you wish me to clarify any detail. If you have questions later, please ask me or another member of staff.*

**Observation process, content and participant selection**

*In order to evaluate our programme of NCD services in Irbid we would like to observe how the group counselling is provided. We will not be asking you any questions. We are randomly choosing which counselling sessions to observe. This observation of services will contribute to our understanding of current services. We will use the findings to help with evaluating and improving the NCD services, research and scientific publication by MSF.*

**Confidentiality and anonymity**

*The observation will be completely confidential and anonymous. We will not be taking any names or making any recordings. We will take notes during our observation, but we will not write down your name. The notes will only be seen by the research team and translator. The notes will be kept privately and anonymously so that no one can link anything you say in the interview back to you. The notes will be securely stored and only accessible by approved study team members. In the future, the anonymised data (without any identifying information about you) may be made available to other researchers in a public data store.*

**Voluntary participation and right to withdraw**

*Participating in the observation is entirely voluntary. You are completely free to not allow the observation to take place. You can also withdraw your consent at any time before OR during the observation, and also afterwards by not allowing any notes to be used by contacting the MSF clinic staff (Hashim Taani: irbid-liaison@oca.msf.org). In this event, the notes will be withdrawn from the database.*

**Risks and benefits of participation**

*It will not directly benefit you in any way but the study findings will benefit the NCD programme users and staff and will be shared with the Jordanian MoH, MSF and the wider global health community through publication.*

*Refusal to participate will not harm you in any way. The choice that you make will have no bearing on the care you receive.*

**Study approvals**

*MSF and the Jordanian Ministry of Health have given their approval for the observation to be conducted. If you have any questions, you can ask them now or later.*

*If you have any questions later, you may contact any of the following:*

**Study Contact Details**

| **Name** | **Position** | **Phone Number** | **Email address** |
| --- | --- | --- | --- |
| Dr Éimhín Ansbro | Principal Investigator, LSHTM |  | eimhin.ansbro@lshtm.ac.uk |
| Dr. Majed Asad | Head of NCD Directorate, Ministry of Health | +962 79 574 0531 | [majedasad@yahoo.com](mailto:majedasad@yahoo.com) |
| Hashim Taani | Research Coordinator, MSF, Irbid | +962 79 749 6191 | [irbid-liaison@oca](mailto:irbid-liaison@oca.msf.oca) |

**Part 2: Certificate of Consent (Observation)**

**Statement by the researcher/person taking consent**

*I have provided the information sheet, accurately read out the information sheet to the potential participant, and to the best of my ability made sure that the participant understands the following:*

1*. Participation is voluntary and can be withdrawn at any time. Refusal to participate will not affect medical care in anyway.*

*2. Data may be withdrawn up to a certain point in the analysis, will be anonymised and is only accessible to the research team*

*3. Study findings will be shared with the participants, MoH, MSF and broader global health community through publication.*

*I confirm that the participant was given an opportunity to ask questions about the study, and all the questions asked by the participant have been answered correctly and to the best of my ability. I confirm that the individual has not been coerced into giving consent, and the consent has been given freely and voluntarily.*

***A copy of this ICF has been provided to the participant.***

*Name of Researcher/person taking the consent_______________________________(Print)*

*_______________________________(Signature)*

*Date ___________________________ (Day/month/year)*

# Annex 2: Data collection forms

## Annex 2a: Topic guide – Focus group discussions with NCD patients

| Key area | Themes | Question |
| --- | --- | --- |
| Introduction | Study aim and agencies involved | Why invited to participate? Consent & any questions? |
| Participant Background | Getting to know each other + building rapport | Could you tell us a bit about yourself? *Prompt: e.g. profession, what area live in, when you were first diagnosed with [NCD condition]?* |
| Reach | Access  Barriers to accessing care for NCDs  Ways of reducing barriers | What do you know about your NCD condition(s)? *Prompt – e.g. causes, types, who gets it, treatment*  Could you tell me about how you came to learn about your NCD condition(s)? ? *Prompt – e.g. from friends/family, from the radio (or other media), when diagnosed at hospital.*  What were you told about NCD condition(s) when you were enrolled in the MSF clinic? *Prompt – probe understanding of NCD condition(s) such as causes, risks and its management (medication and diet).*  What do you think might prevent people from attending this service for their NCD condition(s)? *Prompt: lack of knowledge, lack of services, costs, time, quality of services, stigma etc.*  How could access to healthcare for NCD condition(s) be improved? |
| Adoption and implementation | Information  Support | How did you feel when you were enrolled into this clinic? *Prompt: counselling/support experience. Prompt: subsequent days/weeks experience*  Who did you talk to about your experience at the clinic? *Prompt: E.g. family members, friends.*  What were you told about managing your NCD condition(s) after you were enrolled in clinic (by the NCD staff)? *Prompt: medicine types and usage, managing medicines, diet changes, risks and symptoms, frequency of check-ups etc.*  What sources of support did you receive in managing your NCD condition? *Prompt: emotional support from family/friends, information support from health workers, MHPSS from health workers.*  What made it easier for you to access care – initially and continuing care?  What made it difficult for you to access care – initially and continuing care?  What made it easier for you to self-manage your NCD condition at home?  What made it difficult for you to self-manage your NCD condition at home?  How acceptable do you find the NCD service / treatment. *Prompt: e.g. logistically, socially, culturally etc., differences with previous experience of treatment/ service?*  What has been your experience of the psychosocial services offered by the programme – group sessions/individual counselling*?* |
| Maintenance | Challenges  Supportive factors  To support adoption and implementation | What have been the main challenges in maintaining your medical treatment for your NCD condition? *Prompt: time, costs, information, drug supply, pill burden, stigma/shame etc.*  What have been the main challenges in altering your diet? *Prompt: information, costs, support*  What have been the main challenges in increasing your levels of exercise? *Prompt: information, suitable facilities or locations, physical condition, support, costs*  What have been the main challenges in reducing or quitting smoking? *Prompt: information, support, costs, desire*  What could have made accessing care easier for you? *Prompt: e.g. information given – content and way it was delivered; costs; type and quality of care and support; focus on role of the NCD programme/services.*  What could have made achieving lifestyle changes easier for you? *Prompt: e.g. information given – content and way it was delivered; costs; type and quality of care and support; focus on role of the NCD programme/services;*  What support is available to help you to continue to attend the clinic and self-manage your condition?  What additional supports regarding your NCD condition would you like to have? |
| Effectiveness | Unintended consequences  Benefits | What have been the negative consequences of taking NCD treatment / attending the service? *Prompt: physical, psych, costs, time.*  What have been the benefits of receiving NCD treatment / attending the service? *Prompt: e.g. physical, psychological, social, economic.*  What have been the benefits or negative consequences of attending group sessions/ individual counselling? |
| Thanks and  close | Anything else to add  Questions/Thanks, feedback info | Anything else to add on topic that we haven’t discussed today? Any questions for me? Feedback again on how the discussion will be used and fed back. |

## Annex 2b: Topic guide – Semi-structured interviews with NCD patients

The topic guides for the semi-structured interviews are indicative and they will be refined based on findings from the focus groups.

| Key area | Themes | Question |
| --- | --- | --- |
| Introduction | Study aim and agencies involved  Why invited to participate  Consent & any questions? |  |
| Participant Background | Getting to know each other + building rapport | Could you tell us a bit about yourself? *Prompt: e.g. profession, what area live in, when you were first diagnosed with NCD condition?* |
| Reach | Knowledge in community  Access to testing for [NCD condition]  Barriers to testing | What do you know about your condition? *Prompt – e.g. causes, types, who gets it, treatment*  What has been your experience in accessing healthcare and medications for your condition? *Prompt – in Syria, in Jordan, other NGOs or clinics, why choose to come to MSF clinic, does experience differ?*  Do you think a lot of people have your NCD condition in your community?  What do you think prevents people from accessing healthcare/medications for NCD conditions?  What do you think would make it easier for people to access healthcare / medications for NCD conditions?  If MSF were not providing this service what would you do to manage your condition? |
| Adoption and implementation | Information and other support provided  Adjusting to condition | What type of information provided to you about your condition and its treatment when you were enrolled in the MSF clinic? *Prompt: causes, who gets it, chronic nature, medicine types and usage, managing medicines, diet changes, risks and symptoms, frequency of check-ups etc.*  What sources of support did you receive? *Prompt: emotional support from family/friends, information support from health workers, psychosocial support from health workers*  What other support would you have liked to receive?  Do you find it easy to come in to the clinic from the beginning? *Prompt: facilitators or barriers e.g. logistically, socially, culturally*  How has having your NCD condition changed your daily life/routine? *Prompt: e.g. difficulties in changing your daily routine, in Syria or in Jordan?*  What is your experience of the MHPSS part of the programme (group sessions or individual counselling)? |
| Maintenance | Barriers/challenges to adhering to appointments and prescribed medicine/ lifestyle change. | Do you come in regularly for all your appointments? Do you find it easy or difficult to do so? Why? (e.g. travel, time, stigma)  What is your experience when you come to the clinic?  Do you experience any difficulties when you are visiting the clinic for follow up?  What could be done to make it easier for you to come to the clinic?  Do you take your medicines as often as you are prescribed? Why? *(Prompt: don't think it's important, unsure how to take them, can’t read the instructions, too many pills, share with family/ friends, supply rupture)* Do you find it easy or difficult to do so? Why? (*Prompt* *difficult to remember)*  Do you feel any pressure not to take your medicines (*Prompt: stigma from family or community, cost, medication sharing)*  What could be done to make it easier for you to take your medications?  Do you find it easy to maintain the recommended diet, exercise levels, smoking cessation for your condition?  What has helped you to make lifestyle changes? Prompt: health education, medical staff, family or community support?  What challenges do you face in adapting your diet, exercise levels and smoking habits?  Do you thing MHPSS is important?  What challenges do you face in taking part in or attending MHPSS support (group sessions, or individual counselling)? |
| Effectiveness | How coming to the clinic has affected patient's condition | What have been the negative consequences of taking treatment for the condition/ attending the service? *Prompt: physical, psych, costs, time.*  What have been the benefits of receiving treatment for your condition / attending the service? *Prompt: e.g. physical, psychological, social, economic.*  *What have been the positive and negative consequences for you in attending the MHPSS sessions (group or individual counselling)? (Prompt: feel supported, feel better, assists with managing NCD condition, upsetting, difficult)* |
| Thanks and  close | Anything else to add  Questions/Thanks, feedback info |  |

## Annex 2c: Topic guide – Semi-structured interviews with NCD health care providers and staff

The topic guides for the semi-structured interviews are indicative and they will be refined based on findings from the patient focus groups.

| Key area | Themes | Question |
| --- | --- | --- |
| Introduction | Study aim and agencies involved  Why invited to participate  Consent & any questions? |  |
| Participant Background | Getting to know each other + building rapport | Could you tell us a bit about yourself? Prompt: e.g. professional, involvement in the NCD service at Irbid (and previously if relevant)? |
| Reach | Access  Barriers to NCD care provision  Ways of reducing barriers | What are the key challenges for patients to access healthcare (medications, regular clinical review, investigations, interventions) for their NCD condition(s) e.g. knowledge, costs, time, availability or quality of care [expand], stigma etc.  How could access to healthcare for NCD condition be improved? Prompt: improve knowledge (e.g. outreach, radio, health workers etc.), improve availability of services, quality of services etc. |
| Adoption and implementation | Information and support | What types of information are provided to patients when they are enrolled in/ attend the NCD service?  What sources of support are offered to patients when they are enrolled in/ attend the NCD service)?  How acceptable do you think the MSF NCD programme, including treatment, is for patients? Prompt: e.g. quality, responsiveness, socially, culturally etc.  What is your experience with implementing the new MSF NCD guideline?  What sources of support and information were available to you to facilitate implementing the guideline? |
| Maintenance | Challenges  Supportive factors  To support adoption and implementation | What do you think are the main challenges facing NCD patients here in terms of managing their condition? Prompt: medicines/testing/attendance - time, costs, information, drug supply etc.; lifestyle changes – knowledge, social/cultural pressures etc.  What could be done to make it easier for NCD patients to access care? Prompt: e.g. information given – content and way it was delivered; costs; type and quality of care and support; [note: focus on role of the [NCD condition] programme/services].  What do you think are the main challenges facing staff here in terms of delivering the NCD care programme in Irbid? Prompt: time, training, clinical support/supervision, guidelines or tools  What could be done to make it easier for staff to deliver this NCD care programme? Prompt: knowledge, time, training, clinical support/supervision, guidelines or tools  What are the benefits of using the MSF NCD guideline?  What are the challenges around using the MSF NCD guideline?  What could be done to facilitate implementation of the guideline? |
| Effectiveness | Unintended consequences  Benefits | What are the benefits of the NCD care programme in Irbid? Prompt: more efficient, less complications, for patients, for staff, for system, for community etc.  What are negative consequences of the NCD care programme in Irbid? Prompt: time, complexity, costs etc. for patients, for staff, for system, for community  What particular aspects of the programme have helped or hindered NCD care? Prompt: clinical aspects, task shifting, introduction of HLO, MHPSS, HV, structures, tools, systems  What particular aspects of the guideline have helped or hindered NCD care? Prompt: supports decision making, ease of use, contradictory, not acceptable to patients, different to usual practice in Jordan. |
| Thanks and  close | Anything else to add  Questions  Thanks, feedback info | Anything else to add on topic that we haven’t discussed today?  Any questions for me?  Feedback again on how the discussion will be used and fed back. |

##

## Annex 2d: Topic guide – Semi-structured interviews with key stakeholders

The topic guides for the semi-structured interviews with key stakeholders are indicative and they will be refined based on findings from the patient focus groups and interviews and will vary depending on respondent type.

| Key area | Themes | Question |
| --- | --- | --- |
| Introduction | Study aim and agencies involved  Why invited to participate  Consent & any questions? |  |
| Participant Background | Getting to know each other + building rapport | Could you tell us a bit about yourself? Prompt: e.g. professional, involvement in the NCD service at Irbid (and previously if relevant)? |
| Reach | Access  Barriers to NCD care provision  Ways of reducing barriers | What are the key challenges for patients to access healthcare (medications, regular clinical review, investigations, interventions) for their NCD condition(s) e.g. knowledge, costs, time, availability or quality of care [expand], stigma etc.  How could access to healthcare for NCD condition be improved? Prompt: improve knowledge (e.g. outreach, radio, health workers etc.), improve availability of services, quality of services etc. |
| Adoption and implementation | Information and support | How acceptable do you think the MSF NCD programme, including treatment, is for patients? Prompt: e.g. quality, responsiveness, socially, culturally etc. |
| Maintenance | Challenges  Supportive factors  To support adoption and implementation | What do you think are the main challenges facing NCD patients in this area in terms of managing their condition? Prompt: medicines/testing/attendance - time, costs, information, drug supply etc.; lifestyle changes – knowledge, social/cultural pressures etc.  What could be done to make it easier for NCD patients to access care? Prompt: e.g. information given – content and way it was delivered; costs; type and quality of care and support; [note: focus on role of the [NCD condition] programme/services].  What do you think are the main challenges facing staff here in terms of delivering the NCD care programme in Irbid? Prompt: time, training, clinical support/supervision, guidelines or tools  What could be done to make it easier for staff to deliver NCD care programme? Prompt: knowledge, time, training, clinical support/supervision, guidelines or tools  What are the benefits of using the MSF NCD guideline? [ask if appropriate]  What are the challenges around using the MSF NCD guideline? [ask if appropriate]  What could be done to facilitate implementation of the guideline? [ask if appropriate] |
| Effectiveness | Unintended consequences  Benefits | What are the benefits of the NCD care programme in Irbid? Prompt: more efficient, less complications, for patients, for staff, for system, for community etc.  What are negative consequences of the NCD care programme in Irbid? Prompt: time, complexity, costs etc. for patients, for staff, for system, for community  What particular aspects of the programme have helped or hindered NCD care? Prompt: clinical aspects, task shifting, introduction of HLO, MHPSS, HV, structures, tools, systems  What particular aspects of the guideline have helped or hindered NCD care? Prompt: supports decision making, ease of use, contradictory, not acceptable to patients, different to usual practice in Jordan. |
| Thanks and  close | Anything else to add  Questions  Thanks, feedback info | Anything else to add on topic that we haven’t discussed today?  Any questions for me?  Feedback again on how the discussion will be used and fed back. |

## Annex 2e: Clinical audit sample checklist

|  | Condition | YES | NO | N/A |
| --- | --- | --- | --- | --- |
| 1.0 | Diabetes | | | |
| 1.1 | Micro-albuminuria or urinary protein tested in the last year (Target = 80%)  ? | Y | N | N/A |
| 1.2 | Correct action taken based on micro-albuminuria test result? (Target = 80%)  ? | Y | N | N/A |
| 1.3 | Baseline CVD risk score done on enrolment? | Y | N | N/A |
| 1.4 | Prescribed statin in accordance with guideline (if risk score > 20% or known CVD)? |  |  | N/A |
| 1.5 | On ACE inhibitor (ACEi) inhibitor for HTN if no contraindication (Target 80%)  ? | Y | N | N/A |
| 1.6 | Creatinine tested prior to ACEi initiation? (Target 80%) | Y | N | N/A |
| 1.7 | Annual foot check done? (Target 80%) | Y | N | N/A |
| 1.8 | Correct foot score calculated? (Target 80%) | Y | N | N/A |
| 1.9 | Correct action taken based on foot score? (Target 80%) | Y | N | N/A |
| 1.10 | Annual eye check done or referral made? (Target 80%) | Y | N | N/A |
| 2.0 | Hypertension | | | |
| 2.1 | Annual glucose check done if not known diabetic? (Target 80%) | Y | N | N/A |
| 2.2 | Correct action taken if above target (diagnose diabetes or perform second test as per guideline)? (Target 80%) | Y | N | N/A |
| 2.3 | Annual CVD risk score done? (Target 80%) | Y | N | N/A |
| 2.4 | Annual cholesterol checked if not prescribed a statin? (Target 80%) | Y | N | N/A |
| 3.0 | Asthma | | | |
| 3.1 | Annual control review done (Target = 80%) | Y | N | N/A |
| 3.2 | Action taken following control review as per guideline i.e. step down/up on medications as appropriate? (T 80%) | Y | N | N/A |
| 3.3 | Inhaler technique check documented? (Target 80%) | Y | N | N/A |
| 4.0 | Referral | | | |
| 4.1 | Referral to acute care/specialist service appropriate according to guideline? (T 80%) | Y | N | N/A |

## Annex 2f: Patient self-report medication adherence and beliefs questionnaire

| **Section 1** | |
| --- | --- |
| **1.1** | **Age (tick one):**  16-19 20-29 30-39 40-49 50-59  60-69 70-79 80-89 90-99 |
| **1.2** | **Gender (tick one):** Male Female |
| **1.3** | **Marital status (tick one):**  Single Married Widow(er) Divorced |
| **1.4** | **Household size (tick one box from (a) and one box from (b)) :**  (a) How many people aged 16 or older live with you currently, including non-relatives.  0 1-2 3-4 5-6 > 6 |
| **1.5** | (b) How many children under 16 years of age live with you currently, including non-relatives.  0 1-2 3-4 5-6 > 6 |
| **1.6** | **Highest education level reached (tick one):**  None Primary Secondary High School University |
| **1.7** | **Diagnosis (tick all that apply):**  Diabetes Asthma Chronic Lung Disease (other than asthma)  Thyroid Hypertension Ischaemic Heart Disease/ Angina  Peripheral Vascular Disease Stroke / Transient Ischaemic Attack  Other Please specify: _____________________________________________________________________  ________________________________________________________________________________________ |
| **1.8** | **Please indicate the number of REGULAR medications on your most recent prescription**  **from the MSF clinic** (include tablets or insulin only; exclude equipment e.g. glucose strips etc. )  0 1 2 3 4  6 7-10 11-15 16-20 > 20 |
| **1.9** | **Do you take medications that you get from other sources?** Yes No |
| **1.10** | **If Yes, (circle all that apply):**  Pharmacy Family/friends NGO clinic MOH clinic Private clinic |

| **Section 2**    [copyright material removed] | | | | | | |
| --- | --- | --- | --- | --- | --- | --- |
|  | | | | | | |
|  |  |  |  |  |  |  |
|  |  | | | | | |
| MARS_5VA RH2002  Medication Adherence Report Scale _5 ©R Horne University of Brighton, 1999. | | | | | | |

| **Section 3**  [copyright material removed] |
| --- |
| The Beliefs About Medicines Questionnaire – Specific (BMQ-S11-plural). © R Horne, University of Brighton, 1996. |

| **4.1** | TO BE ANSWERED BY MSF STAFF MEMBER  Questionnaire filled by (circle on option): | Patient 1  MSF Staff 2 |
| --- | --- | --- |

1. <http://journals.plos.org/plosone/article?id=10.1371/journal.pone.0065174> [↑](#footnote-ref-1)
